# Cryptic variation fuels plant phenotypic change through hierarchical epistasis

**DOI:** 10.1038/s41586-025-09243-0

**Published:** 2025-07-09

**Authors:** Sophia G. Zebell, Carlos Martí-Gómez, Blaine Fitzgerald, Camila P. Cunha, Michael Lach, Brooke M. Seman, Anat Hendelman, Simon Sretenovic, Yiping Qi, Madelaine Bartlett, Yuval Eshed, David M. McCandlish, Zachary B. Lippman

**Affiliations:** 1. Cold Spring Harbor Laboratory, Cold Spring Harbor, NY, USA.; 2. Howard Hughes Medical Institute, Cold Spring Harbor Laboratory, Cold Spring Harbor, NY, USA.; 3. Department of Plant and Environmental Sciences, Weizmann Institute of Science, Rehovot, Israel.; 4. Department of Plant Science and Landscape Architecture, University of Maryland, College Park, MD, USA.; 5. Sainsbury Laboratory Cambridge University (SLCU), Cambridge, UK

**Keywords:** cryptic mutations, paralogs, redundancy, cis-regulatory, tomato, inflorescence, gene regulatory network, modeling, epistasis

## Abstract

Cryptic genetic variants exert minimal phenotypic effects alone but are hypothesized to form a vast reservoir of genetic diversity driving trait evolvability through epistatic interactions^1–3^. This classical theory has been reinvigorated by pan-genomics, which is revealing pervasive variation within gene families, cis-regulatory regions, and regulatory networks^4,5,6^. Testing cryptic variation’s capacity to fuel phenotypic diversification has been hindered by intractable genetics, limited allelic diversity, and inadequate phenotypic resolution. Here, guided by natural and engineered cis-regulatory cryptic variants in a paralogous gene pair, we identified additional redundant trans regulators, establishing a regulatory network controlling tomato inflorescence architecture. By combining coding mutations with cis-regulatory alleles in populations segregating for all four network genes, we generated 216 genotypes spanning a wide spectrum of inflorescence complexity and quantified branching in over 35,000 inflorescences. Analysis of this high-resolution genotype-phenotype map using a hierarchical model of epistasis revealed a layer of dose-dependent interactions within paralog pairs enhancing branching, culminating in strong, synergistic effects. However, we also uncovered an unexpected layer of antagonism between paralog pairs, where accumulating mutations in one pair progressively diminished the effects of mutations in the other. Our results demonstrate how gene regulatory network architecture and complex dosage effects from paralog diversification converge to shape phenotypic space, producing the potential for both strongly buffered phenotypes and sudden bursts of phenotypic change.

## INTRODUCTION

An enduring debate in evolutionary biology concerns the extent to which small-effect genetic variants contribute to expanding phenotypic diversity from developmentally stabilized (canalized) states^7–10^. The most enigmatic cohort among small-effect variants may be cryptic alleles^1,2^. In their simplest form, cryptic alleles have substantial effects on phenotypes only through interactions with environmental factors or with other alleles, including other alleles that may themselves be phenotypically cryptic^3^. Although hidden at the level of organismal phenotype, cryptic alleles can generate molecular phenotypes through altering protein function or gene expression–and are most likely to accumulate and remain hidden in buffered molecular contexts, such as redundancy within gene families and gene regulatory networks^11^. The accumulation of cryptic alleles in buffered contexts may be a major source of genomic variation shaping network architecture and trajectories of phenotypic evolution. Under this hypothesis, epistatic interactions between previously cryptic alleles may result in the sudden appearance of phenotypic variation in previously invariant traits facilitating both within-species adaptation and macroevolutionary transitions^11–13^.

Demonstrating cryptic variation’s contribution to trait evolution is challenging. Genetic dissection of trait variation is typically confined to single species or a few closely related ones, where only narrow ranges of phenotypic diversity can be assessed. Moreover, most dissections of natural trait variation expose only major effect variants, as is also true for developmental genetics in model systems, leaving the influence of cryptic alleles on natural populations and gene regulatory networks largely unexplored^1,14^. Importantly, background dependencies–likely stemming in part from cryptic alleles–are common in evolutionary and developmental genetics^15–18^. Yet, despite these clues, efforts to systematically dissect cryptic variation and its role in phenotypic evolution remain hampered by the complex and often poorly characterized structure and redundancy of gene regulatory networks, limited allelic diversity, and restricted phenotypic resolution in most systems.

Genome editing in model systems with complex developmental programs offers a powerful approach to interrogate cryptic variation. Beyond applications in medicine and agriculture, genome editing enables the engineering of customized mutations and allelic series with wide ranges of phenotypic effects in isogenic backgrounds^19^. This capability allows for deep exploration of gene function and interactions among different classes of mutations, including *cis*-regulatory variants and paralog duplications and losses that influence dosage but are often cryptic^20–23^. While pairwise interactions are often detectable, how diverse allelic variants interact across larger regulatory and developmental networks remains unexplored. Here, we use genome editing in tomato (*Solanum lycopersicum*) to interrogate cryptic variation in a genetic network.

## RESULTS

**Fig. 1:**
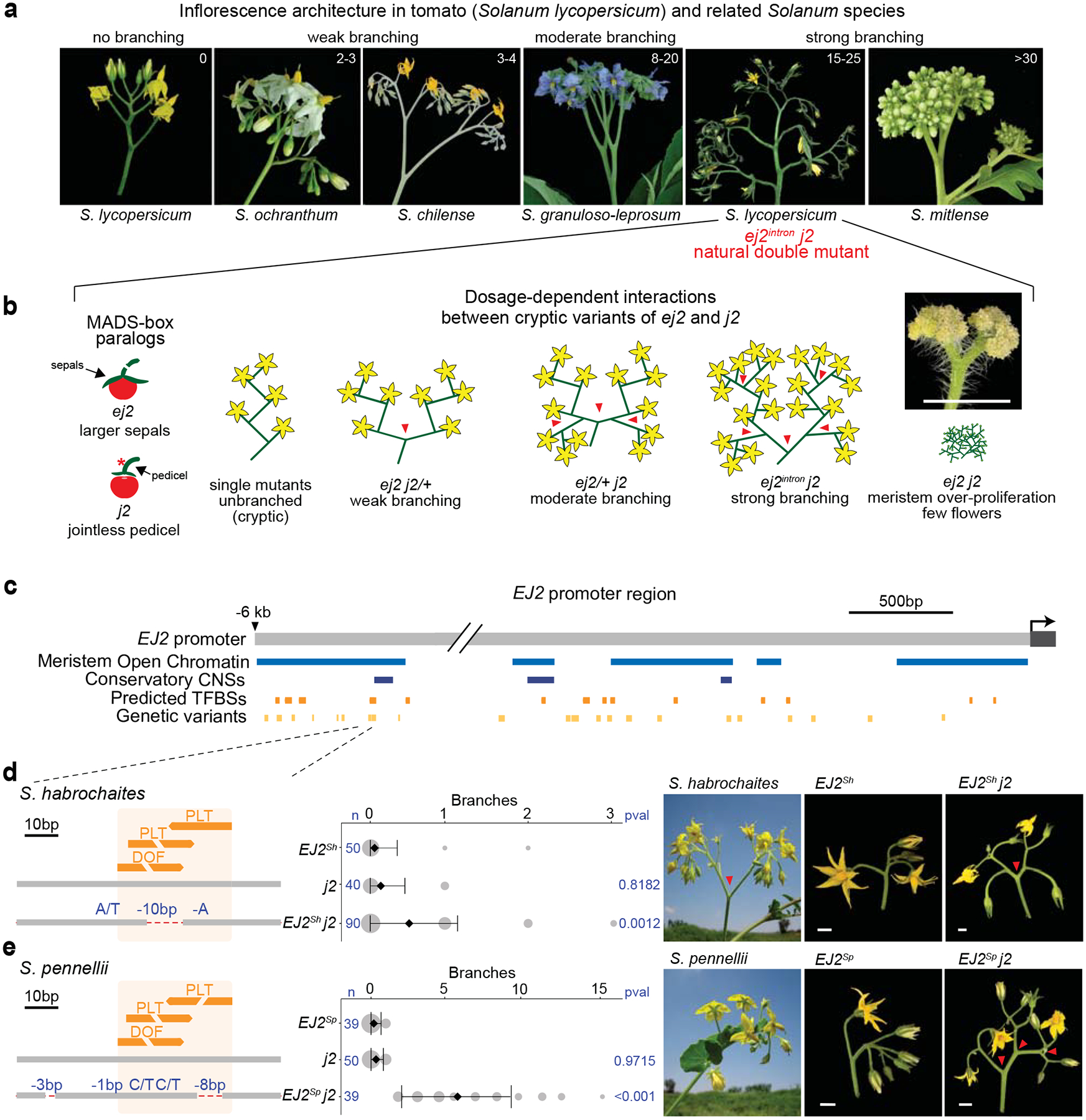
Natural *cis*-regulatory variants of the *SEP* gene *EJ2*. **(a)** Increased complexity of inflorescences of *Solanum* species. **(b)** Dose-dependent redundancy relationship among the SEP paralogs *EJ2* and *J2* in controlling tomato inflorescence architecture. Inset: Proliferated *ej2 j2* meristem, scale bar is 1 cm. **(c)** A 6 kb region upstream of the *EJ2* transcription start site showing open chromatin (blue), conserved non-coding sequences (CNSs, dark blue), predicted transcription factor binding sites (TFBSs, orange), and pan-genome variants (light orange). **(d)** *Cis*-regulatory region of *S. lycopersicum* and *S. habrochaites EJ2* with one DOF (DOMAIN OF UNKNOWN FUNCTION) and two PLT (PLETHORA) TFBSs. The overlapping DOF-PLT site is disrupted by a 10 bp deletion in *S. habrochaites*. Quantification of inflorescence branching for the three indicated genotypes (middle) is followed by representative images of *S. habrochaites*, the *EJ2*-containing introgression line (LA3925, *EJ2*^*Sh*^), and *EJ2*^*Sh*^ in the *j2* background (*EJ2*^*Sh*^ *j2*, right). **(e)** *Cis*-regulatory region of *S. pennellii* showing disruption of all three TFBSs by a 10 bp deletion and linked SNVs, quantification of inflorescence branching for the three indicated genotypes (middle) and representative images of *S. pennelli*, the *EJ2*-containing introgression line (IL3-4, *EJ2*^*Sp*^), and *EJ2*^*Sp*^ in the *j2* background (*EJ2*^*Sp*^ *j2*, right). For ‘d’ and ‘e’: Dashed red lines, deleted sequences; Blue text, deletion sizes and SNVs. Area of grey circles, numbers of inflorescences quantified; black diamonds and bars, mean values and standard deviations. Total number of inflorescences (n) and p-values from Two-sided Dunnett’s Compare with Control Test. Red arrowheads mark branch points. Scale bars are 1 cm.

Natural variation in the architectures of plant reproductive branching systems (inflorescences) within the Solanaceae family, particularly in the *Solanum* genus, exemplifies how evolutionary processes generate morphological diversity (Fig. 1a)^24,25^. Cryptic variation may shape such trait diversity, such as by limiting possible phenotypic states, making tomato an ideal system to test this hypothesis within a controlled genetic framework. Many tomato mutants—often involving cryptic genetic interactions—mirror natural variation across the family, offering a platform to systematically dissect how cryptic variation can influence inflorescence architecture^26^. We uncovered epistasis between cryptic natural coding and *cis*-regulatory variants in two MADS-box transcription factor genes of the *SEPALLATA* (*SEP*) clade, which have conserved roles in inflorescence development (Fig. 1b)^27,28^. Interactions between mutations in the *SEP* gene *JOINTLESS2* (*J2*), originating from the wild species *S. cheesmaniae*, and a natural *cis*-regulatory variant in its paralog *ENHANCER OF JOINTLESS2* (*EJ2*), result in highly branched inflorescences through classical redundancy epistasis^27^. While individual mutations in each paralog are cryptic on inflorescence branching, different combinations of homozygous and heterozygous genotypes produce varying degrees of branching effects, reflecting a dose-dependent epistatic relationship (Fig. 1b). Notably, while *EJ2* is conserved across the Solanaceae, *J2* is absent in many species and cultivated genotypes (Supplementary Fig. 1), making these species sensitive to changes in inflorescence architecture from variations in *EJ2* dosage^4^. The *J2*-*EJ2* paralog relationship offers a system to study how epistatic interactions between cryptic alleles in a regulatory network influence trait evolvability. However, realizing this potential first requires identifying additional network components.

### Cryptic variants of MADS-box gene *EJ2*

To examine whether natural variation in the epistatic relationship between *J2* and *EJ2* could further contribute to inflorescence architecture diversity, we mined tomato pan-genome data for variation in the *EJ2* promoter^5,6^. Through overlap with open chromatin in tomato reproductive meristems and predicted transcription factor binding sites (TFBSs) (Fig. 1c)^29^, we filtered 629 candidate variants of greater than 5 base pairs (Supplementary Table 1, Supplementary Fig. 2) down to two small deletions with nearby single-nucleotide variants (SNVs) that coincided with a cluster of three TFBSs located 6 kbp upstream of *EJ2* (Fig. 1c,d, Supplementary Table 2). These variants were found only in the wild species *S. habrochaites* and *S. pennellii*, which produce weakly branched inflorescences (Fig. 1d,e).

**Fig. 2:**
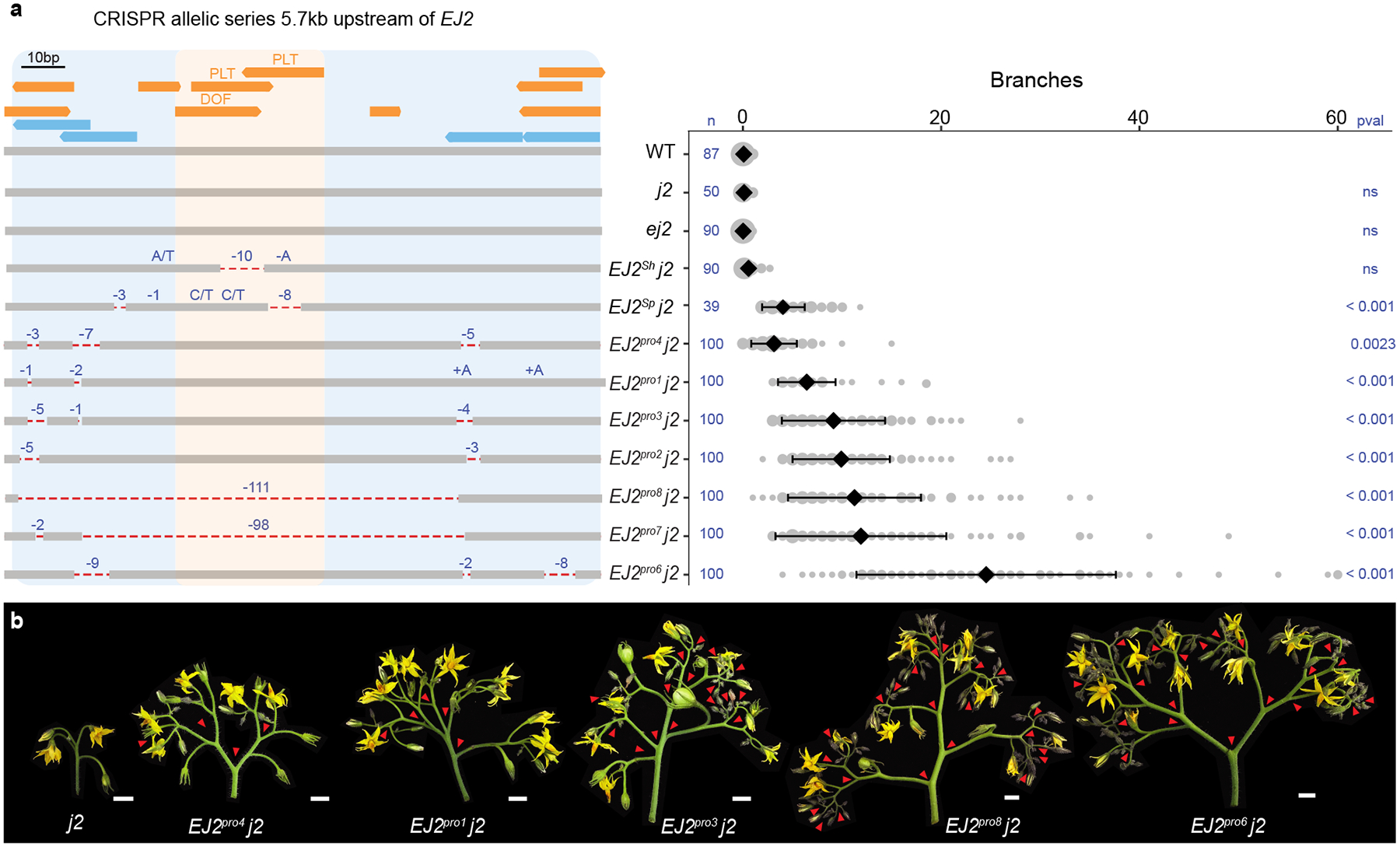
Engineered *EJ2 cis*-regulatory alleles pinpoint a branching specific enhancer. **(a)** Left: 153 bp target region located 5.7kb upstream of the *EJ2* translation start site showing annotated TFBSs (orange), including the focal *DOF* and *PLT* binding sites, and four CRISPR/Cas9 gRNAs (light blue) used for generation of the *EJ2* promoter (*EJ2*^*pro*^) allelic series. Two lines with strong phenotypes (*EJ2*^*pro7*^ *j2* and *EJ2*^*pro8*^ *j2*) carried overlapping ~100 bp deletions that removed the *DOF-PLT* binding sites, as well as other lower confidence predicted binding sites (Supplementary Table 2, 26). Dashed red lines, deleted sequences; Deletion sizes and SNVs, blue. Right: Quantifications of inflorescence branching for each genotype. Area of grey circles, numbers of inflorescences; black diamonds and bars, mean values and standard deviations. Total number (n) of observed inflorescences and p-values from Two-sided Dunnett’s Compare with Control Test. **(b)** Representative images of *j2* and five of the *EJ2*^*pro*^ alleles in the *j2* background, capturing the range of branching effects. Red arrowheads mark branch points. Scale bars are 1 cm.

Tomato introgression lines carrying wild species chromosomal segments with these *EJ2* variants in isogenic backgrounds (LA3925, designated *EJ2*^*Sh*^, and IL3-4, *EJ2*^*Sp*^) rarely exhibit branching (Fig. 1d,e). However, branching increased with the addition of the *j2* mutation. *EJ2*^*Sh*^ *j2* plants exhibited a subtle but significant increase in branching compared to the *EJ2*^*Sh*^ genotype (Fig. 1d), and *EJ2*^*Sp*^ *j2* plants produced an average of five branches per inflorescence (Fig. 1e, Supplementary Tables 3–4). Since introgression lines have large chromosomal segments carrying additional variants, we used CRISPR to test whether branching specifically resulted from TFBS disruption by attempting to create similar deletions in *j2* mutant background. Due to the absence of Cas9 gRNA target sites in the 25 bp region, we first used the more permissive Cas9 SpRY variant, which recognizes an expanded protospacer-adjacent motif (PAM)^30,31^. This approach, which used three gRNAs and catalytically active and dead versions of Cas9 fused to an adenine base editor, produced three alleles, each with a single SNV within one TFBS. As none of these single nucleotide changes led to branching (Supplementary Fig. 2a,b, Supplementary Tables 5, 6), we targeted a 153 bp target region flanking the TFBSs using four conventional Cas9 gRNAs. We recovered seven *EJ2* promoter (*EJ2*^*pro*^) alleles (Fig. 2a): five with small indels and SNVs, often at the gRNA target sites, and two with overlapping ~100 bp deletions spanning the entire interval. Notably, none of these alleles exhibited branching in the functional *J2* background, but all caused branching in the mutant *j2* background, displaying a continuous range of effects (Fig. 2b, Supplementary Tables 7, 8). Additionally, none of these genotypes exhibited the pleiotropic phenotypes observed in loss of function double mutants, such as enlarged sepals or altered fruit shape (Supplementary Fig. 3, Supplementary Tables 9–12)^27^. We targeted two additional promoter regions with open chromatin or sequence conservation located 1.6 kbp and 2.1 kbp upstream. Four deletion alleles of varying sizes were generated in each region, but only those affecting the second region, including a ~3.3 kbp deletion, produced mild branching (1–5 branches on average; Supplementary Fig. 2, Supplementary Tables 13–16). These findings indicate that several promoter segments regulate *EJ2*, and the TFBSs disrupted in *EJ2*^*Sh*^ and *EJ2*^*Sp*^, together with nearby sites removed in our engineered alleles, act collectively to positively regulate *EJ2* expression and control inflorescence development.

### PLT paralogs regulate *EJ2* and branching

Our finding that multiple *cis*-regulatory cryptic alleles caused branching, partly due to the disruption of the TFBSs affected by two natural alleles, prompted us to investigate whether the transcription factors predicted to bind these sites directly regulate *EJ2* expression and inflorescence architecture. Both the *S. habrochaites* and *S. pennellii EJ2 cis*-regulatory alleles are predicted to disrupt binding sites for the DOMAIN OF UNKNOWN FUNCTION (DOF) and the PLETHORA (PLT) transcription factor families. Members of these families have been implicated in meristem development in *Arabidopsis thaliana* (PLT)^32^ and flowering in tomato (DOF)^33^ Using our tomato inflorescence development expression atlas^34^, we searched for *DOF* and *PLT* genes expressed during key developmental stages. Among the 34 *DOF* genes in tomato*, SlDOF9* emerged as a primary candidate (Supplementary Fig. 4), because engineered mutants of this gene develop more flowers on inflorescences with weak branching^33^. However, our CRISPR mutants exhibited a drastic change in leaf shape but did not show branching, either alone or in the *j2* background (Supplementary Fig. 4, Supplementary Tables 17,18).

**Extended Data Figure 1:**
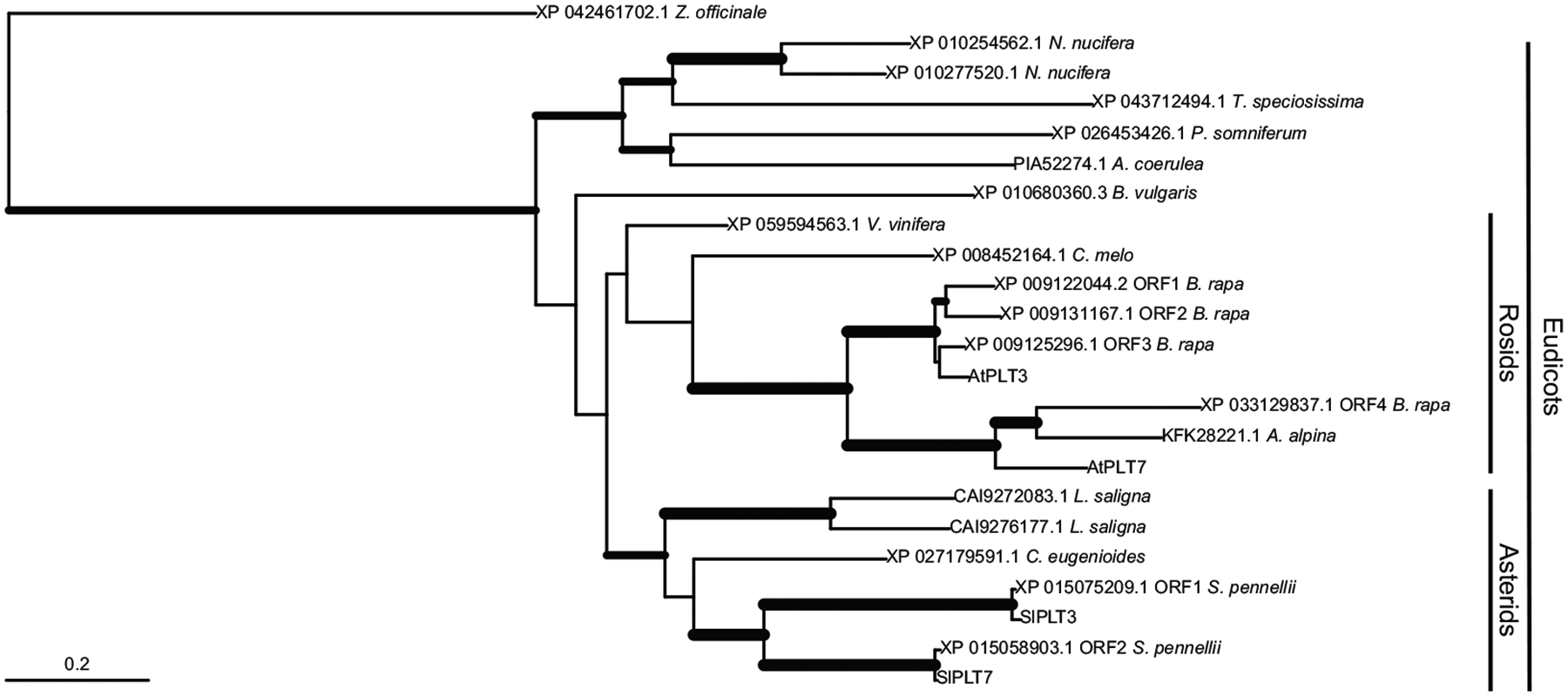
Phylogenetic tree of PLT3/PLT7. Phylogenetic tree of PLT3 and PLT7 proteins from species selected for taxonomic breadth, representing asterids, rosids, early eudicots, and monocots, see Methods for details. Branch thickness reflects bootstrap support: thin lines indicate support <75, medium lines 75–90, and thick lines >90. Scale bar represents number of amino acid substitutions per site.

**Fig. 3:**
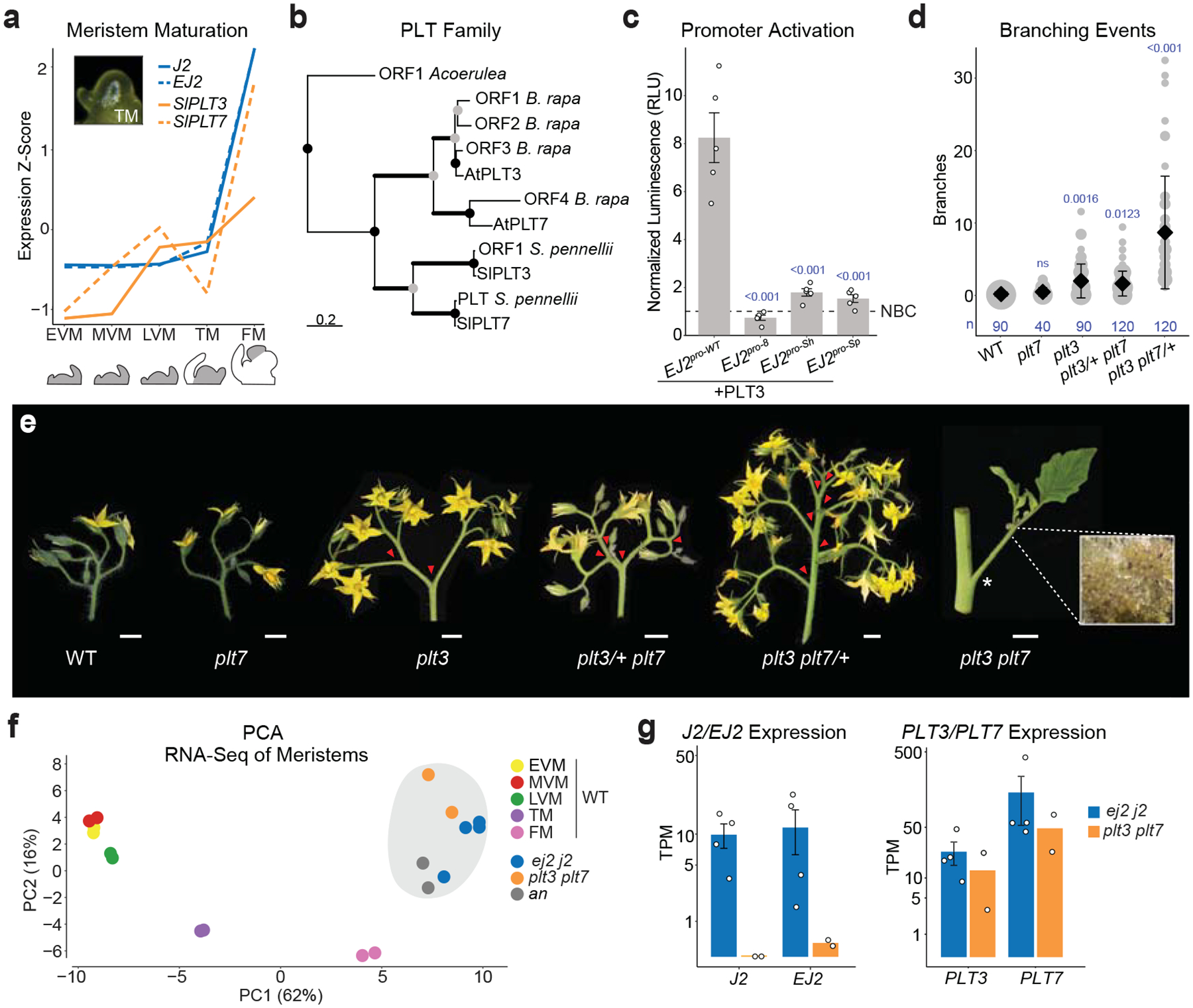
*PLETHORA* transcription factor paralogs control inflorescence architecture upstream of *EJ2/J2*. **(a)** Expression dynamics of *EJ2, J2, PLT3*, and *PLT7* over five developmental stages (grey): Early Vegetative Meristem (EVM), Middle (MVM), Late (LVM), Transition (TM), and Floral Meristem (FM). **(b)** Pruned phylogenetic tree of PLT3 and PLT7 proteins. Branch thickness reflects bootstrap support: thin lines indicate support <75, medium lines 75–90, and thick lines >90. Scale bar represents the number of amino acid substitutions per site, presumed duplication and speciation nodes denoted by grey and black circles, respectively. (for full phylogeny see Extended Data Fig. 1). **(c)** *In planta* heterologous expression of firefly luciferase driven by wild type and *EJ2 cis*-regulatory alleles, co-transfected with PLT3 and normalized to an internal constitutive renilla luciferase and nonbinding protein controls (NBC), open circles represent individual biological replicates, n= 5, error bars represent standard error and p-values from Two-sided Dunnett’s Compare with Control Test shown in blue. **(d)** Quantification of branching in double *plt3/7* mutants, including single and homozygous/heterozygous combinations. Grey circles correspond to the number of inflorescences, black diamonds are mean values and bars are standard deviations. Number (n) of observations and p-values from Two-sided Dunnett’s Compare with Control Test shown in blue. **(e)** Representative inflorescences of *plt3* and *plt7* mutant combinations. Inset is double mutant inflorescence showing meristem over-proliferation (5x magnification). Red arrowheads mark branch points. Scale bars are 1 cm. **(f)** Principal Component Analysis of top 200 genes differentially expressed over meristem maturation in staged WT meristems and proliferating meristems of the mutant genotypes: *ej2 j2, plt3 plt7*, and *an*. **(g)** Expression of paralogs *EJ2, J2, PLT3*, and *PLT7* in proliferating meristems of the mutants *ej2 j2* and *plt3 plt7* on a log(1+ transcripts per million) scale, error bars represent standard error, n = 4 samples for *ej2 j2*, 2 samples for *plt3 plt7*.

We next focused on two closely related *PLT* paralogs (*SlPLT3* and *SlPLT7;* hereafter *PLT3* and *PLT7*), expressed in meristems during and after floral transition, similar to *J2* and *EJ2* (Fig. 3a). These tomato *PLT* paralogs are orthologs of Arabidopsis *AtPLT3* and *AtPLT7* but arose from independent duplications (Fig. 3b, Extended Data Fig. 1). In Arabidopsis, *AtPLT3* and *AtPLT7* function in meristem maturation as well as floral organ identity and growth^35^. We tested whether the PLT proteins bind the *EJ2* promoter and activate its expression by performing a heterologous dual-luciferase assay in tobacco (*Nicotiana benthamiana*) leaves. Although the full-length coding sequence of *PLT7* could not be cloned or synthesized, PLT3 strongly activated the intact *EJ2* promoter but not the *EJ2*^*pro8*^, *EJ2*^*pro-Sh*^ and *EJ2*^*pro-Sp*^ alleles, which have mutated PLT binding sites (Fig. 3c, Supplementary Tables 19, 20). For normalization, we included ETHYLENE RESPONSIVE12 (ERF12) as a non-binding control, a transcription factor expressed in meristems with a single DNA-binding domain structurally similar to those in PLTs.

We mutated both *PLT* paralogs using CRISPR/Cas9. *plt7* single mutants appeared wild type, while *plt3* mutants produced inflorescences with few branches. However, double mutants exhibited extreme meristem overproliferation and branching. These mutant alleles also displayed dose-dependent redundancy: *plt3/+ plt7* genotypes showed weak branching, while *plt3 plt7/+* genotypes exhibited moderate branching (Fig. 3d,e, Supplementary Table 21, 22). The binding assays, combined with the quantitative effects of the *plt* mutant genotypes and the intermediate branching observed in all *EJ2pro j2* genotypes, suggest that the PLTs transcriptionally regulate *EJ2* and likely other genes involved in inflorescence development. This aligns with the presence of PLT binding sites in the *cis*-regulatory regions of *J2* (Supplementary Table 23).

To further characterize the functional relationships between the *PLT* and *SEP* paralogs, we profiled and compared gene expression in proliferating meristems from the two double mutants. A principal component analysis (PCA) of the top 200 maturation marker genes from wild type meristem stages^34^ revealed that both double mutants cluster closest to the floral meristem maturation stage and also with the *anantha* (*an*) mutant, which overproliferates floral meristems due to a mutation in the ortholog of the Arabidopsis *UNUSUAL FLORAL ORGANS* gene (Fig. 3f). The expression data also showed that *J2* and *EJ2* are very low expressed in *plt3 plt7* meristems, whereas both *PLTs* remain expressed in *j2 ej2* meristems, supporting regulation of these *SEPs* by the PLTs (Fig. 3f,g).

### A *PLT-SEP* genotype-phenotype map

**Fig. 4:**
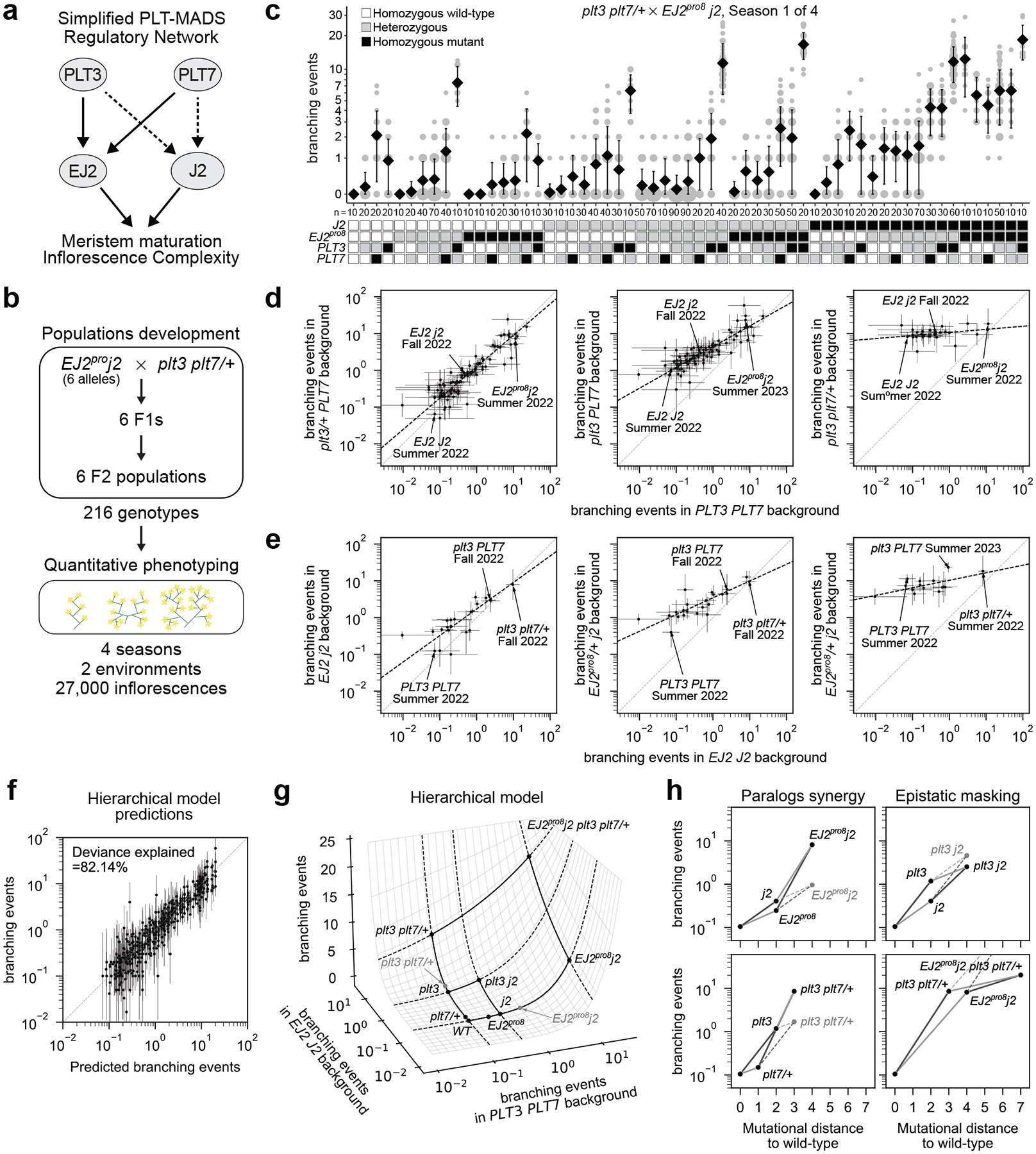
A hierarchical model of genetic interactions explains *PLT-SEP* inflorescence genotype-phenotype map. **(a)** Simplified architecture of the *PLT3/7-EJ2/J2* genetic and molecular network. Arrows represent positive regulation. Solid arrows are supported by genetic and molecular evidence. Dashed arrows are supported by annotated binding sites. **(b)** Six F2 populations with different *EJ2*^*pro*^ alleles were phenotyped across multiple seasons and environments, yielding observations of per-inflorescence branching events for 216 different genotypes. **(c)** Representative data for inflorescence branching from a single season of one of six populations, log(1+x) scale. Area of grey circles, number of inflorescences, black diamonds and bars, mean values and standard deviations. Total number (n) of observations. Genotypes represented as wild-type (white), mutant (black), or heterozygous (gray). **(d, e)** Maximum Likelihood Estimates (MLEs) for mean number of branching events for combinations of mutations in *EJ2* and *J2* (d) or *PLT3* and *PLT7* (e) vs. wild-type across different genetic backgrounds of mutations in *PLT3* and *PLT7* (d) or *EJ2*^*pro8*^ and *J2* loci (e). Black dashed lines represent total least squares regression lines. n=10 inflorescences per plant; see Methods for details. **(f)** Comparison of the predicted number of branching events by the hierarchical model and the MLE for each genotype-season combination. Error bars represent 95% confidence intervals. Genotype-season combinations with 95% confidence intervals wider than a thousand-fold range are not shown (d-f). **(g)** Representation of the inferred hierarchical epistasis model, where the surface shows how phenotypes from perturbing each paralog pair in the wild-type background are combined to determine the average phenotype of genotypes with mutations in both pairs. Grey dots represent the multiplicative expectation for the designated within-paralog mutant combinations. **(h)** Predicted branching events under the hierarchical model in specific genotypes as a function of their mutational distance to the wild-type. Grey dots indicate the multiplicative expectation.

Our genetic and molecular analyses identified key components of an inflorescence regulatory network comprising two duplicated transcription factor pairs with dose-dependent epistatic interactions. We generated multiple alleles for these factors in a shared isogenic background, including both promoter regulatory site alleles and strong coding alleles, all of which are cryptic individually. This genetic resource enabled us to systematically explore the phenotypic space and quantify the functional output arising from variations within a simple, rapidly evolving network involving the interactions among *PLT3* and *PLT7*, and their downstream targets, *J2* and *EJ2*. (Fig. 4a).

To explore the genotype-phenotype map of this tomato inflorescence regulatory network, including the interplay between cryptic dosage effects and paralogous epistatic relationships, we selected six *EJ2*^*pro*^ *j2* lines spanning a range of branching effects and crossed them with *plt3 plt7/+* plants to generate six F2 segregating populations (Fig. 4b, Methods). These populations provided 216 genotypic combinations, enabling an in-depth phenotypic and statistical analysis of branching effects from single, double, and higher-order mutants, as well as dosage effects from heterozygosity (Fig. 4c). Across four field seasons in two environments, we quantified a total of 35,606 inflorescences (Supplementary Table 24). Preliminary analyses indicated greater variance in the number of branching events per inflorescence both within plants and within genotypes than would be expected if branching events were Poisson distributed (Supplementary Figure 5a,b). Consequently, we treated branching events as overdispersed count data in all subsequent analyses, which provided a significantly improved fit relative to a Poisson error model (likelihood ratio test for negative binomial versus Poisson: p < 10^−16^; Methods). For illustration, data from a single population and field season are shown in Fig. 4c, with the full dataset available in Supplementary Table 24.

Using the data from this large set of crosses, we sought to determine how mutations within this genetic network combine to determine the mean number of branching events per inflorescence for any given genotype. We began by comparing a model where mutations at different loci combine additively versus a model where mutations at different loci combine multiplicatively (with both models accounting for dominance interactions within each locus). The model with multiplicative effects fit substantially better (59.40% deviance explained by an additive model with a log link versus 46.05% deviance explained by an additive model with an identity link Supplementary Fig. 5c,d), suggesting an overall tendency for mutations at different loci to interact multiplicatively in this system. Using this multiplicative model as a baseline, we then fit a more complex model to determine whether epistatic interactions between loci are also present. We found that a pairwise interaction model that included these epistatic interactions significantly outperformed the multiplicative model (83.11% deviance explained, likelihood ratio test: p < 10^−16^) and achieved greater predictive performance for held-out seasons and genotypes (Supplementary Fig. 6; average leave-one-out cross-validated R^2^ on held-out seasons was 0.89 for the pairwise model compared to 0.70 for the multiplicative model). This pairwise model detected pervasive epistasis across loci, with 43 of 80 epistatic terms showing p-values < 0.05 (Supplementary Table 25). Among the most notable of these interactions were strong, super-multiplicative, synergistic interactions between *plt3* and *plt7* as well as between the *EJ2*^*pro*^ alleles and *j2*, such that combining mutations within a paralog pair often results in a mean number of branching events several fold (3- to 9) in excess of what would be expected when multiplying the effects of the individual mutations (Table 1).

**Extended Data Figure 2:**
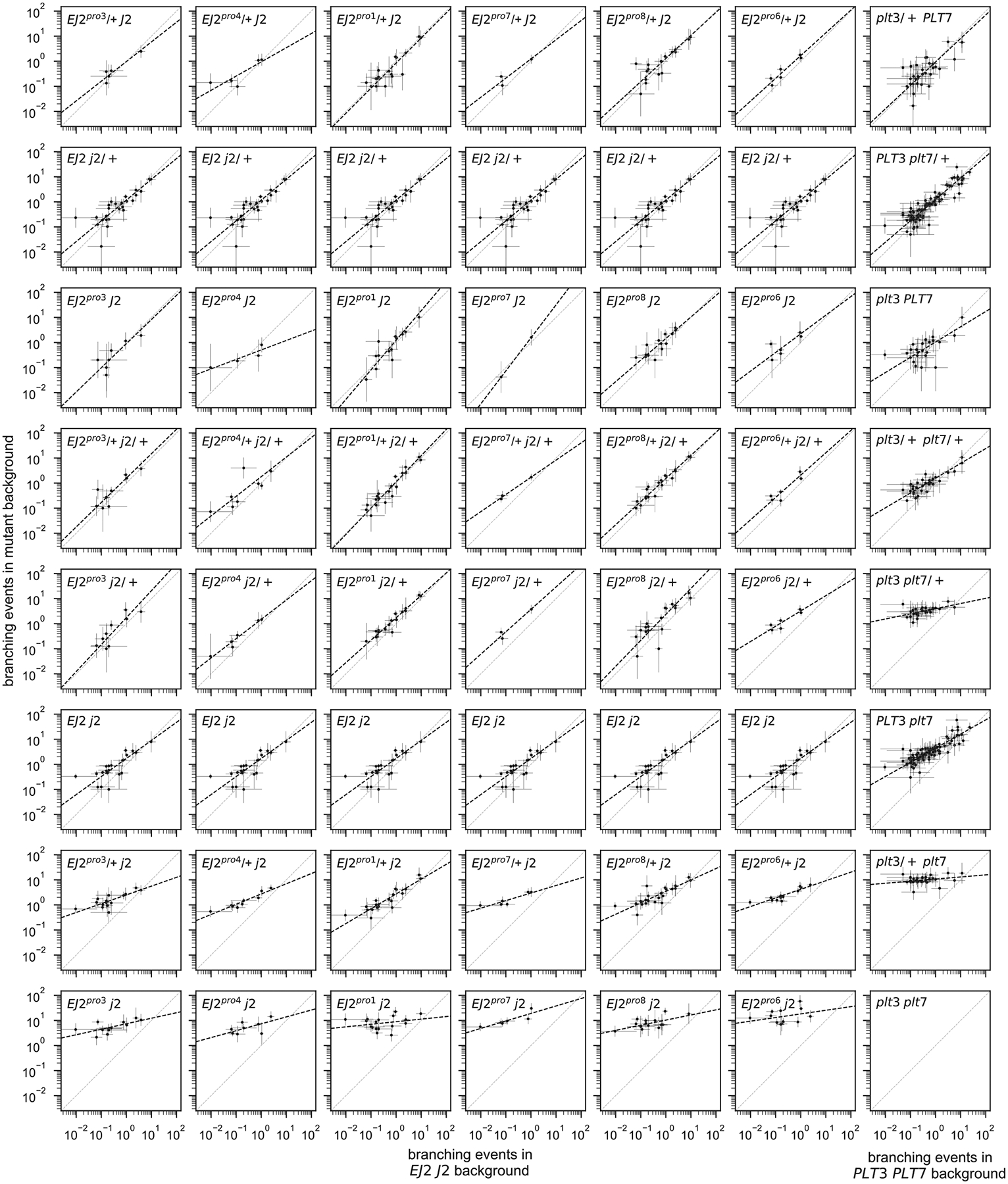
Mutations in one paralog pair linearly re-scale the effects of mutations in the other paralog pair. Scatterplots representing the expected number of branching events. Every dot represents a possible combination of mutations in *PLT3* and *PLT7* (first 6 columns) or *EJ2* and *J2* (last column) in a specific season. The value plotted on the y-axis corresponds to the phenotype conferred by the given combinations of mutations either in the designated background at *EJ2* and *J2* (first 6 columns) or the designated background at *PLT3* and *PLT7* (last column); x-axis values are given by the phenotype of each set of mutations in the wild-type background. Dots represent the Maximum Likelihood Estimate (MLE) of the expected number of branching events for genotypes in specific seasons, as estimated under a negative binomial model. Error bars represent the 95% confidence interval for the MLEs. Total least squares regression lines for the log MLEs are represented with black dashed lines. Genotype-season combinations with a 95% confidence interval wider than a thousand-fold range are not shown.

**Extended Data Figure 3:**
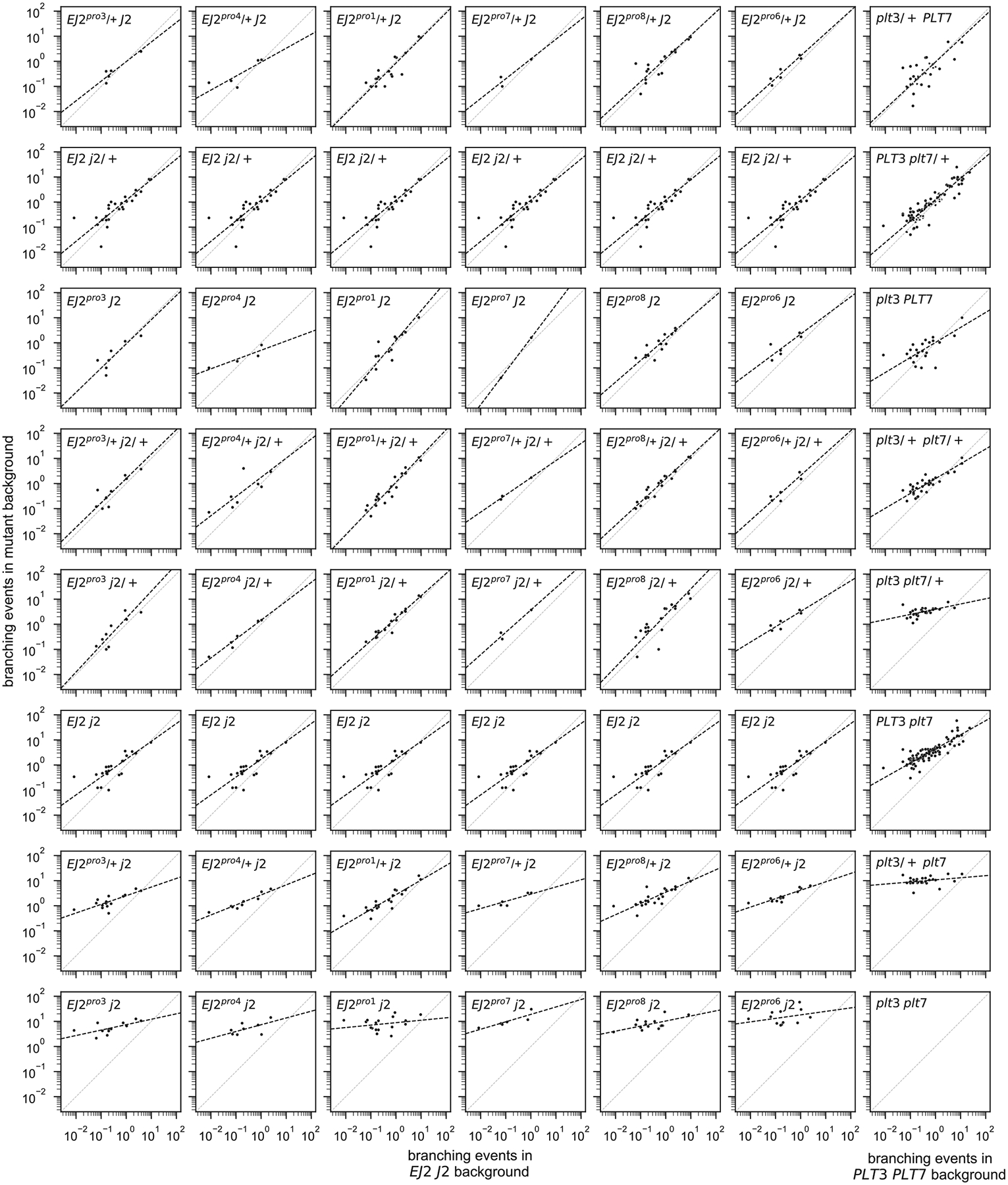
Linear rescaling of mutational effects observed between paralog pairs is robust under an alternative method for estimating genotype-season means. Scatterplots representing the sample average number of branching events. Every dot represents a possible combination of mutations in *PLT3* and *PLT7* (first 6 columns) or *EJ2* and *J2* (last column) in a specific season. The value plotted on the y-axis corresponds to the sample average phenotype conferred by the given combinations of mutations either in the designated background at *EJ2* and *J2* (first 6 columns) or the designated background at *PLT3* and *PLT7* (last column); x-axis values are given by the sample average of each set of mutations in the wild-type background. Total least squares regression lines for the log sample means are represented with black dashed lines. Genotype-season combinations in which no branching was observed are not shown.

While we observed strong positive interactions within paralog pairs, consistent with our previous observation regarding at least partial redundancy, we also inferred many negative interactions between non-paralogous pairs (11 out of 14 additive-by-additive epistatic terms between non-paralogous pairs were negative and showed p-values < 0.05, Supplementary Table 25). To better understand the structure of the genotype-phenotype relationship implied by these negative interactions, we first calculated maximum likelihood estimates for the average number of branching events per inflorescence across the 470 genotype-season combinations in our data set. We then plotted how the log-transformed phenotypes conferred by different combinations of *j2* and *EJ2*^*pro*^ mutations are transformed when placed on different *plt3* and *plt7* mutant backgrounds as compared to a wild-type *PLT3* and *PLT7* background. Under a multiplicative model of interaction across paralog pairs, these quantities are expected to be linearly related with a slope of exactly one and an intercept that varies with the strength of the genetic perturbation at the *PLT3* and *PLT7* loci. Interestingly, while we observed that the relationship between backgrounds is always linear, the slope of this linear relationship was not constant, but decreased as mutations accumulated in the *PLT3* and *PLT7* loci (Fig. 4d, Extended Data Fig. 2). We observed the same pattern when analyzing the phenotypes resulting from *plt3* and *plt7* mutant combinations across different *j2* and *EJ2*^*pro8*^ backgrounds, with slopes decreasing as the strength of the *EJ2 J2* perturbations increased (Fig. 4e). Importantly, this pattern was consistent across all six *EJ2*^*pro*^ alleles (Extended Data Fig. 2) and remained robust across other methods of estimating average phenotype (Extended Data Fig. 3). Collectively, these analyses suggest a surprisingly simple form of genetic interaction between the paralog pairs, where mutations in one paralog pair linearly re-scale the effects of mutations in the other. As the slope of the linear relationship reduces almost to zero in highly mutated genetic backgrounds, the negative interaction coefficients between the paralog pairs reflect a systematic pattern of masking interactions between non-paralogous mutations, wherein the effects of mutations in one pair is diminished when the other pair is highly mutated.

### Modeling hierarchical epistasis

**Extended Data Figure 4:**
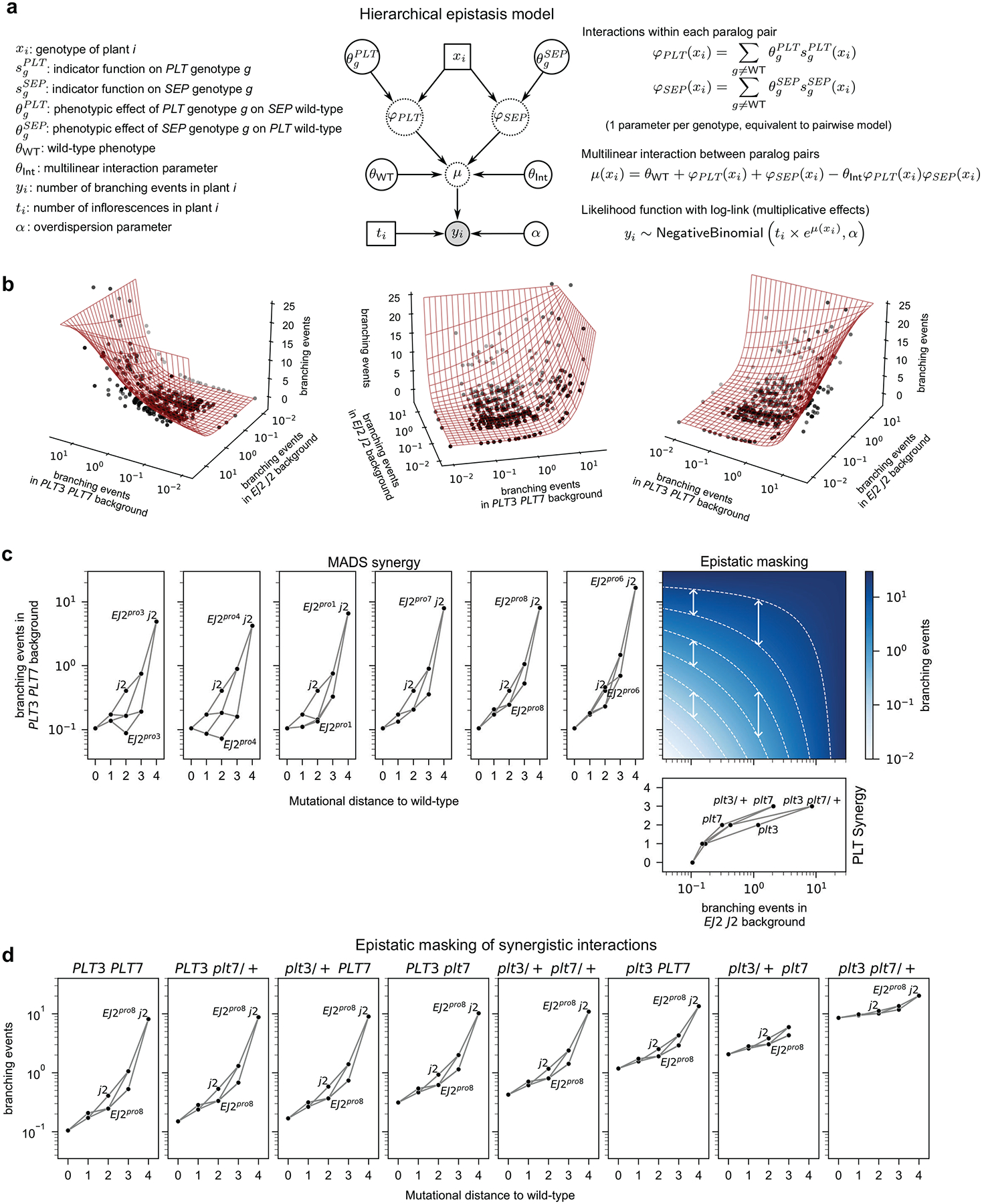
Understanding synergy and masking under the inferred hierarchical model. **(a)** Graphical representation of the hierarchical epistasis model. Equations on the right show the mathematical transformations applied at each layer. See Methods for details and an explanation of the mathematical notation. **(b)** Different views of the two-dimensional surface representing the expected number of branching events as a function of the mean branching events conferred by placing the combination of mutations in each paralog pair in the wild-type background of the other pair. Points represent the independent maximum likelihood estimates (MLEs) for the expected number of branching events for the measured genotype-season combinations. Error bars represent the 95% confidence interval for the MLEs. n=10 inflorescences per plant; see Methods for details. **(c)** Complete representation of the inferred hierarchical model of gene interaction. Left and bottom panels represent the estimated number of branching events for each paralog pair combination in the wild-type background of the other pair as a function of the Hamming distance to the wild-type genotype. Different *EJ2*^*pro*^ alleles show phenotypic effects of different magnitude but all interact with *J2* in a similar way. The phenotype for any genotype is obtained by combining the independent effect of the mutations in each pair of paralogs through the two-dimensional surface in the middle panel. This heatmap represents the inferred multilinear function that quantitatively characterizes epistatic masking between PLT and MADS genes. White dashed lines represent isophenotypic lines, this is, combinations of background phenotypic effects that result in the same number of branching events when combined. Note that the function is linear across any horizontal or vertical transect, as illustrated by the constant distance between isophenotypic lines across any transect. Deviation from a slope of −1 in the shape of the isophenotypic lines, which are scale-independent, indicates the presence of an epistatic interaction between the two pairs of paralogs. Note that distance between isophenotypic lines increases in highly mutated backgrounds, indicating that a larger perturbation is required to achieve the same phenotypic outcome. **(d)** Estimated number of branching events for all *EJ2*^*pro8*^ *J2* combinations across genetic backgrounds with an increasing number of mutations in *PLT3* and *PLT7* illustrates how the synergistic interactions between *EJ2*^*pro8*^ and *j2* become masked as *PLT3* and *PLT7* become increasingly mutated.

This mode of genetic interaction, in which each mutation systematically re-scales the effects of other mutations, is a defining feature of a classic theoretical model of epistasis known as the multilinear model^36^. To simultaneously capture the super-multiplicative synergistic interactions within paralog pairs together with the systematic masking interactions between paralog pairs, we thus fit an additional model that we call the hierarchical epistasis model because it treats this multilinear interaction between paralog pairs as an additional layer of epistasis. In this model, similar to the pairwise model, we allowed an arbitrary pattern of dominance and epistasis within each paralog pair, but the predictions based on the coefficients for each pair of paralogs are combined according to a multilinear interaction. Crucially, because for a multilinear interaction mutations in one of the pairs of paralogs simply rescale the effects of mutations in the other pair, this multilinear interaction between paralog pairs uses only a single parameter to describe the extent to which mutations in one paralog pair mask the effects of mutations in the other pair, in contrast to our more traditional pairwise interaction model that uses 42 parameters to describe interactions between the paralog pairs (i.e. additive-by-additive, dominance-by-dominance, and additive-by-dominance parameters for each of the 14 non-paralogous pairs of mutations). Finally, after the within-paralog pair interactions are combined across paralog pairs using a multilinear interaction, the results are transformed once more using an exponential function (i.e. we again use a log link, identical to that used for the pairwise model; see Methods and Extended Data Fig. 4a for additional technical details on the hierarchical epistasis model). We applied this hierarchical epistasis model to our data and found it recapitulated the observed phenotypic measurements nearly as well as the full pairwise model (82.14% deviance explained, Fig. 4f), and maintained high predictive power for held-out data from complete seasons and unobserved genotypes (Supplementary Fig. 6, average leave-one-season-out cross-validated R^2^=0.89), despite the substantially reduced number of parameters. Using the Akaike Information Criterion (AIC) to compare the two models^37,38^, we found that the hierarchical model is roughly 18,358 times more likely than the pairwise model (AIC_hiearchical_ - AIC_pairwise_ = −19.64).

To provide a more intuitive understanding of the behavior of this hierarchical model, it is useful to visualize the combination of interaction between paralog pairs and the exponential mapping as a response surface^39^ that depicts the predicted phenotype of a combination of mutations across both paralog pairs as a function of the phenotypes conferred when mutating each paralog pair separately (Fig. 4g, Extended Data Fig. 4b). In this response surface, the phenotypes conferred by within-pair mutations are shown on a log scale, so that the effects of mutations that combine according to the multiplicative expectation would add along each axis. However, consistent with the pairwise model, interactions within each paralog pair show a strong pattern of synergy (Fig. 4h, left, see Extended Data Fig. 4c for all other within paralog pair interactions), such that double mutants are substantially further displaced along each axis than would be expected from the individual effects of the single mutants (Fig. 4g, multiplicative expectation shown by gray dots). The key difference from the pairwise model is that the interactions between paralog pairs, instead of being represented by numerous between-pair interaction terms, are given by a surface (Fig. 4g) whose shape is controlled by a single parameter that determines the overall strength of the interaction between the paralog pairs, and which has horizontal and vertical transects that are exponential due to the final exponentiation (Extended Data Fig. 4c). In particular, we see that as the phenotypic effect of one paralog pair increases, the corresponding transect for mutations in the other pair becomes progressively flatter, reflecting diminishing phenotypic contributions from additional mutations (Extended Data Fig. 4d). While this masking effect is modest for mutations with moderate phenotypic impacts (e.g., the 11.24-fold effect of a homozygous *plt3* mutation in a wild-type background is reduced to 6.18-fold in a *j2* background, Fig. 4h, **top right**), it becomes far more pronounced in highly mutated backgrounds (e.g., adding *plt3 plt7/+* to a wild-type *EJ2 J2* background has an 81.30-fold effect, but only a 2.49-fold effect in the *EJ2*^*pro8*^ *j2* background, Fig. 4h, **bottom right**). Overall, these results show how synergy within gene families can result in an opening of phenotypic space in which previously cryptic mutations have strong effects, but also show how this expansion of phenotypic space begins to close as accumulated mutations in one part of a genetic network increasingly mask the effect of mutations in other parts.

## DISCUSSION

Here, we used natural and engineered cryptic cis-regulatory variation to identify and functionally dissect transcription factor regulators of tomato inflorescence development in a four-gene regulatory network. Engineering additional cryptic cis-regulatory alleles with continuous epistatic effects and densely sampling thousands of inflorescences from hundreds of combinatorial genotypes allowed us to resolve the genetic architecture and genotype-phenotype map of this network. Mutations within this network tended to interact multiplicatively, but with even stronger positive synergistic (i.e. super-multiplicative) interactions within paralogous gene pairs, consistent with frequent redundancy between paralogs^40,41^. Unexpectedly, we detected dose-dependent masking interactions acting simultaneously between paralog pairs, where mutations in one pair systematically shrink the effects of mutations in the other pair.

Pan-genome sequencing within and across taxa provides a rich resource for understanding how genetic networks are wired. Here, genetic variation in *S. habrochaites* and *S. pennellii* suggested the *PLT*s as candidate regulators of inflorescence morphology in tomato. However, pan-genome sequencing has also revealed extensive and diverse forms of variation whose molecular, developmental, and evolutionary significance remains unknown. Widespread structural variation in cis-regulatory regions and the frequent duplication and loss of both small and large genomic regions drive quantitative expression variance and gene dosage^21,42,43^. The dynamic emergence, divergence, and turnover of paralogous genes can alter the architectures and buffering of regulatory networks, thus perturbing component dosage and potentiating phenotypic change from canalized states when cryptic variants in paralogs converge.

Our detailed dissection of genetic architecture revealed how the *PLT-SEP* network can shift from a canalized state to one poised to release both subtle and substantial phenotypic change. We inferred a hierarchical structure of genetic interactions: classical synergistic interactions within paralog pairs combine via a multilinear interaction between the two paralog pairs and then are transformed once more by an exponential mapping to determine average branches per inflorescence. While our phenomenological modeling is by its nature insufficient to reveal the precise molecular and cellular mechanisms underlying these interactions, we note that each step in our concatenation of simple models is mechanistically plausible, where in particular the partial redundancy within paralog pairs feeds into a regulatory network that requires both *PLTs* and *SEPs* for proper functioning, and where gene action in the context of growing cell populations within a developing meristem provides a plausible basis for the overall tendency of mutations to act multiplicatively. Our finding of a multilinear interaction between the two paralog pairs is notable because, while the multilinear model has been explored theoretically^36,44,45^ and often is used to estimate how directional selection changes additive genetic variance^46,47^, it has received limited empirical support for capturing real-world patterns of epistasis^46,48^. Nonetheless, the multilinear model can be viewed as a continuous relaxation of Boolean (binary ‘on’ or ‘off’) gene regulatory models^49^, accommodating a spectrum of allelic strengths and dosage effects rather than treating gene expression as a binary. We thus hypothesize that synergistic interactions within gene families, combined via multilinear interactions that reflect the structure and functional logic of gene regulatory networks, are likely a common genetic architecture that governs how phenotypic space is simultaneously expanded and constrained.

Notably, the *PLT-SEP* genetic network has accumulated extensive genetic variation both within tomato and between Solanaceae species. *J2* is a relatively recent duplication missing in many Solanaceae^4,28^ (Supplementary Fig. 1). While the *PLT* paralogs are broadly retained, their redundancy relationships have likely diverged and vary between genotypes and species. In contrast, in the Brassicaceae, while *PLT3* and *PLT7* orthologs are conserved, *J2* and *EJ2* MADS-box paralogs have been lost. Interestingly, Arabidopsis *plt* mutants do not have altered inflorescence architecture and branching is constrained across species in the family^50^. The architecture of *PLT-SEP* regulatory networks in the Solanaceae may allow cryptic variation to accumulate more readily than in the Brassicaceae where the *J2/EJ2* subclade is missing, thereby enhancing evolvability of Solanaceae branching. Testing this hypothesis will require broader identification of causal variation across species in these families. Critically, as we show (Fig. 1 and 3), this approach can test specific hypotheses while revealing additional components of conserved and diverged gene regulatory networks. More broadly, the principles uncovered here—where varying paralog redundancy relationships and presence-absence variation shape trait evolvability through genetic interactions that follow the multilinear model—likely extend across regulatory networks underlying other developmental and physiological programs, influencing the evolutionary trajectories of many traits.

Finally, a key aspect of our study was engineered genetic variation that densely sampled genotypic and phenotypic space in a controlled isogenic background. This approach provided the resolution needed to define the character and quantitative form of gene action and epistasis, represented as a surface illustrating how phenotypic effects from mutations within a paralog family combine when incorporating mutations across gene families (Fig. 4g). Detailed mapping of genetic interactions in this way could help reconcile the observation of widespread epistasis in model organisms with the challenge of detecting epistatic effects from allelic variation in natural populations^15–18,51,52^. Placing natural alleles and their combinations onto similar surfaces could reveal how interactions among standing variants push populations into regions of genotypic space where phenotypic variation is either amplified, suppressed, or both. Beyond evolutionary insights, this framework has practical implications in crop engineering^53,54^. Understanding how distinct genetic combinations, along with the specific forms of epistasis they engender, can yield similar phenotypic outcomes may inform targeted editing strategies to predictably ‘tune’ epistatic interactions. By shifting populations or individuals to advantageous positions on the genotype–phenotype surface, such strategies could minimize undesirable pleiotropic effects and circumvent genetic constraints imposed by natural alleles in both complex breeding populations and elite genotypes. Realizing these opportunities will hinge on future research that encompasses larger networks of interacting genes, emphasizing how taxon-specific complements of paralogous genes and their variants shape network architecture—and hierarchical epistasis—across broader evolutionary clades.

## METHODS

### Motif enrichment and variant discovery

FIMO motif enrichment was performed on the sequence of open chromatin regions in the tomato meristem upstream of *SlEJ2* using the *Arabidopsis thaliana* non-redundant motif database curated at Plant TFDB (p-value < 0.00001 and q-value < 0.01)^55^. The same regions were used to search for insertion-deletion (indel) variants called previously from the tomato pangenome^5^. Indels overlapping with annotated motifs were confirmed to not exist in linkage with previously reported *EJ2* variants (*ej2*^*w*^*, sb3*) by PCR and then used for subsequent experiments (see Plant materials) (Supplementary Table 1)^27,56^.

### Plant materials

Seeds of wild type *Solanum lycopersicum* (cultivar M82, LA3475), *Solanum habrochaites* (LA1777), and *Solanum pennellii* (LA0716) were from our stocks. Introgression line IL3-4 (*Solanum pennellii* chromosome 3 introgressed into M82, LA4046) was obtained from the Tomato Genome Resource Center (Department of Plant Sciences, University of California at Davis) and the variant was validated by PCR amplification and Sanger sequencing (all primers used in this study are reported in Supplementary Table 27)^57^. Two overlapping *Solanum habrochaites* chromosome 3 introgression lines LA3925 and LA3926, introgressed into tomato cultivar TA209, were obtained from the Tomato Genome Resource Center (Department of Plant Sciences, University of California at Davis)^58^. Upon validation by PCR amplification and Sanger sequencing, only LA3925 contained the *ShEJ2*^*pro-3*^ variant of interest, so LA3926 was used as a control for crosses between M82 and the introgressed region in the TA209 background. Mutants *j2-TE ej2*^*w*^ and *j2 ej2* were from our stocks, as previously described^27^.

### Genome editing

CRISPR-Cas9 mutagenesis and generation of transgenic tomato plants were performed following our standard protocol^59^. Briefly, guide RNAs (gRNAs) were designed using the Geneious Prime software (https://www.geneious.com/) (gRNAs used in this study are listed in Supplementary Table 27). For Cas9 multiplex editing, the Golden Gate cloning system was used to assemble the binary vector containing the Cas9 and the specific gRNAs^59,60^. For SpRY editing, vectors were constructed through a modular Gateway^™^ assembly, as described previously (Invitrogen)^61^. Final binary vectors were then transformed into the tomato cultivar M82 by *Agrobacterium tumefaciens*-mediated transformation through tissue culture^62^. First-generation transgenic plants (T0) were genotyped with specific primers surrounding the target sites (all primers used in this study are reported in Supplementary Table 27). To purify alleles from potential spontaneous mutations or CRISPR-Cas9 off-target effects following plant transformation, all T0 transgenic lines were backcrossed (BC1) to parental wild-type plants. BC1 populations were then screened by PCR and Kanamycin herbicide susceptibility for plants lacking the Cas9 transgene, PCR products of the targeted regions were Sanger sequenced to confirm inheritance of alleles, and allele-specific genotyping assays were designed for genotyping in subsequent generations. Selected BC1 plants were self-fertilized to generate F2 populations, and these segregating populations were used to validate the phenotypic effects of each allele by co-segregation. F2 or F3 homozygous mutant plants were then used for subsequent crossing and quantitative phenotypic analyses.

### Growth conditions and phenotyping

Seeds were directly sown in soil in 96-cell plastic flats and grown to 4-week-old seedlings in the greenhouse. Seedlings were then transplanted to 4L pots in the greenhouse for crossing and bulking purposes or directly to the fields at Cold Spring Harbor Laboratory, New York or at The University of Florida Gulf Coast Research and Education Center. Greenhouse conditions are long-day (16 h light, 26–28 °C / 8 h dark, 18–20 °C; 40–60% relative humidity) with natural light supplemented with artificial light from high-pressure sodium bulbs (~250 μmol m^−2^ s^−1^). Plants in the fields were grown under drip irrigation and standard fertilizer regimes, and were used for quantifications of inflorescence branching, fruit shape, and sepal length.

To quantify inflorescence branching, inflorescences were counted in order of emergence in two rounds, approximately 60 days post sowing and 75 days post sowing. When available, four primary inflorescences and six axillary inflorescences were counted per plant. 60 or fewer branches were counted, if branching exceeded 60, “too many to count” (“TMTC”) was recorded and the number of branching events was treated as 60 for downstream analysis. Proliferated meristem in the place of inflorescence was indicated in the data as “proliferated”. Occasionally, inflorescences would fail to develop into countable structures, possibly due to stress, in which case, “inhibited” was recorded.

To quantify fruit shape, 10 fruits were collected at mature green stage, cut in transverse sections, and scanned on a single plane. The ratio of maximum height to width, Fruit Shape Index I, was determined from scanned images using Tomato Analyzer^63^. To quantify sepal length, 10 closed mature floral buds of similar developmental stage (1–2 days before anthesis, i.e., before flower opening) per genotype were collected, length of sepals and petals were manually measured and the sepal/petal ratio was calculated^27^.

### Phylogenetic Trees

*J2/EJ2* phylogeny was adapted from Benoit *et al*.^4^. Putative orthologs of SlPLT3/7 and AtPLT3/7 were identified using NCBI BLASTP against proteomes of species selected for taxonomic breadth, representing asterids, rosids, early eudicots, and monocots. Retrieved protein sequences were aligned using MAFFT (v7.505) with default parameters. An HMM profile was constructed from the alignment using hmmbuild in HMMER (v3.3.2) and used to search combined species proteomes with hmmsearch (E-value < 1e-5) to identify additional homologs. All hits were extracted, aligned with MAFFT, and manually trimmed when necessary. A maximum likelihood phylogenetic tree was inferred using IQ-TREE (v2.2.2) with automatic model selection (-m MFP) and 1,000 ultrafast bootstrap replicates (-bb 1000). The resulting tree was rooted using XP_042461702.1_Zofficinale as an outgroup. Bootstrap support values were used to modulate branch thickness in the visualization: branches with support >90 were plotted thickest, those between 75–90 were medium, and those <75 remained thin. The tree was visualized in R using the ape package (v5.8-1).

### RNA extraction and Illumina sequencing

Inflorescence meristems were collected from n = 4 plants at 8 weeks old under stereoscope magnification. Tissue was frozen, ground with beads, and RNA was extracted with TRIzol (Invitrogen) and a Direct-zol RNA Miniprep kit with on-column DNA digestion (Zymo Research). RNA was quantified with Qubit fluorimeter RNA HS assay kit (Invitrogen). Samples were treated with Ribo-Zero rRNA removal kit (Epicenter) and libraries prepared with a TruSeq V2 RNA-Seq prep kit (Illumina).

### RNA Sequencing Analysis

Published RNA-seq data of wild-type M82, *ej2*, *j2*, and *anantha* mutant meristems were downloaded from SRA PRJNA376115, and PRJNA343677^24,34^. Reads were trimmed with Trimmomatic (ILLUMINACLIP:TruSeq2-PE.fa:2:30:10:1:FALSE LEADING:3 TRAILING:3 SLIDINGWINDOW:4:15 MINLEN:36) and aligned to the cDNA annotation of the reference genome sequence of tomato (SL4.0) using STAR v2.6.1.d^64^. Normalization and quantification of individual transcript expression was done in R by calculating transcripts per million (TPM). Differential expression was calculated in R by DESeq2 time course analysis with LRT and the top 200 most differentially expressed genes (log2FC) across wild type meristem maturation were used for principal component analysis of all meristem samples using Python scikit-learn PCA.transform^65^.

### Dual Luciferase assay

A Gateway^™^-compatible dual-luciferase reporter vector (pSZ106) was assembled using the MoClo GoldenGate assembly system^60,66^. Briefly, a Gateway^™^ AttR4-AttL1R cassette (Invitrogen) was cloned upstream of a 46 base pair minimal 35S promoter driving the Firefly luciferase coding sequence (pICSL80001-pL0_fLUC-I (CDS1)) with a Nopaline Synthetase terminator (pICH41421)^60,66^. A Cauliflower Mosaic Virus 35S promoter (pICH51266) was cloned upstream of the coding sequence of Renilla luciferase (pSB123 - pL0_rLUC-I (CDS1), Addgene) with a Nopaline Synthetase terminator (pICH41421)^66^. Both luciferase expression cassettes were cloned into the pICSL4723 binary vector backbone with an NPTII selection cassette. *SlEJ2*^*pro-3*^, *ShEJ2*^*pro-3*^ and *SpEJ2*^*pro-3*^ alleles were cloned into pDONR^™^ P4-P1r and introduced into pSZ106 by Gateway^™^ cloning (Invitrogen). *SlERF12* (Solyc02g077840), *SlPLT380* (Solyc05g051380), and *SlPLT710-short* (Solyc11g010710) were cloned into pDONR207 and introduced into pEAQ-HT-DEST3 by Gateway^™^ cloning (Invitrogen).

All binary expression vectors were transformed into *Agrobacterium tumefaciens* and cultured at 28°C overnight in selective media. Overnight cultures were diluted and grown at 28°C to OD600 1 AU, spun down, and washed into inductive media (10mM MES pH 5.7, 10mM MgCl_2_, 100μM 3’,5’-Dimethyoxy-4’-hydroxyacetophenone) at OD600 1 AU. Bacteria was induced for 3 hours lying horizontally at the bench, then equal volumes of promoter and TF media were combined and co-infiltrated into young fully expanded leaves of 4 week old *Nicotiana benthamiana* plants grown in long days (16 h light / 8 h dark, 22°C; 40–60% relative humidity). Plants were returned to the growth chamber and 100mg tissue was collected and frozen for measurement 3 days after infiltration.

Luciferase activity was measured using a Dual Luciferase Reporter Assay System kit (Promega) according to Moyle *et al*.^67^. Briefly, tissue was homogenized in a Spex Sample Prep 2010 Geno/Grinder (Cole Parmer) and 10 mg of tissue powder was mixed with 100 μl of passive lysis buffer (Promega). Cellular debris was pelleted at 7,500 × g for 1 min, and supernatant was diluted 40X in passive lysis buffer and 15 μl of sample was transferred to 3 replicate wells of a white flat-bottom Costar 96-well plate (Corning). The assay was measured using a GloMax 96 microplate luminometer, and 75 μl per well of luciferase assay reagent and Stop & Glo reagent were added and measured stepwise (Promega).

### Statistics and Reproducibility

All phenotping and molecular experiments were repeated in at least three seasons with similar results. Transcriptomics was performed once with biological replication. For Figures 4e and 4f, n = 10 inflorescences per plant and the number of biologically independent plants per genotype–season combination varies, with quartiles at 2, 5, and 9 plants (source data available in Supplementary Table 24).

### Segregating Populations

BC1 inbred plants of genotype *EJ2pro j2* were crossed to BC1 inbred plants of genotype *plt3 plt7/+* and genotyped in the F1 generation by allele specific PCR to determine the presence of all desired alleles. Segregating F2 seed was sown in the greenhouse in populations of either 192 or 384 plants, tissue was collected for DNA extraction and plants were transplanted without prior genotyping over the course of four seasons, two in fields at Cold Spring Harbor Laboratory, New York and two at The University of Florida Gulf Coast Research and Education Center. Genotypes of plants were confirmed after phenotyping by allele-specific PCR assays.

### Linear regression models

Phenotypic data was summarized at the plant level for quantitative modeling, with abnormal inflorescences marked as “proliferated” or “inhibited” excluded from further analysis. For each plant *i*, we consider the total number of branching events across all inflorescences *y*_*i*_, and the number of inflorescences *t*_*i*_. The total number of branching events *y*_*i*_ was modeled as being either Poisson or negative binomially distributed with exposure *t*_*i*_.

$$y_{i}\sim \text{Poisson}\left({t_{i}\times f}^{-1}\left(\mu \left(x_{i}\right)\right)\right)\;y_{i}\sim \text{NegativeBinomial}\left({t_{i}\times f}^{-1}\left(\mu \left(x_{i}\right)\right),\alpha \right),$$

where f represents a link function and $\mu \left(x_{i}\right)$ represents the phenotypic mean of the genotype $x_{i}$ of plant i, and α is the overdispersion parameter. $\text{Var}\left(y_{i}\right)=\mu \left(x_{i}\right)+\alpha {\mu \left(x_{i}\right)}^{2}$ so α reflects the additional variance relative to the expectation under a Poisson model. Under an additive model, the expected mean $\mu_{\text{add}}(x)$ of any given genotype x is given by:

$$\mu_{add}\left(x_{i}\right)=\theta_{0}+\sum_{l}\theta_{l}^{a}s_{l}^{a}\left(x_{i}\right)+\theta_{l}^{d}s_{l}^{d}\left(x_{i}\right),$$

where $s_{l}^{a}\left(x_{i}\right)=\left\{–1,0,1\right\}$ and $s_{l}^{d}\left(x_{i}\right)=\left\{0,1,0\right\}$ if locus l in genotype $x_{i}$ is homozygous wild-type, heterozygous mutant and homozygous mutant, respectively. Thus $\theta_{l}^{a}$ represents the homozygous effects at locus l, whereas $\theta_{l}^{d}$ represents the deviation of the heterozygous effect from the semi-dominant expectation.

A basis for pairwise interaction models was built by extending the basis for the additive model with additional basis vectors composed by taking the product of each possible pair of additive and dominance components^68^. Under a pairwise model, the expected mean is given by:

$$\mu_{\text{pw}}\left(x_{i}\right)=\mu_{\text{add}}\left(x_{i}\right)+\sum_{l,m}\theta_{l,m}^{a,a}s_{l}^{a}\left(x_{i}\right)s_{m}^{a}\left(x_{i}\right)+\theta_{l,m}^{a,d}s_{l}^{a}\left(x_{i}\right)s_{m}^{d}\left(x_{i}\right)+\theta_{l,m}^{d,a}s_{l}^{d}\left(x_{i}\right)s_{m}^{a}\left(x_{i}\right)+\theta_{l,m}^{d,d}s_{l}^{d}\left(x_{i}\right)s_{m}^{d}\left(x_{i}\right).$$

Models were defined and fit using *statsmodels*^69^ python package using Poisson and negative binomial likelihoods with the identity (f(x)=x) and log link functions (f(x)=logx). Genotype-season Maximum Likelihood Estimates (MLE) for the number of branching events were obtained by defining a dummy variable for each genotype-season combination that took a value of 1 for plants of that genotype and 0 otherwise, while assuming that all genotypes share the overdispersion parameter for the negative binomial likelihood function representing plant-to-plant variability that is jointly estimated with the genotype-season means. Confidence intervals for the MLEs were derived using *statsmodels* with a log-link between model parameters (genotype estimates) and the average number of branching events.

### Hierarchical model

Data was modeled with a negative binomial likelihood function as explained in the Linear regression models section. In this hierarchical model, each paralog pair has an effect that is modeled separately through a complete pairwise interaction model into *φ*_*PLT*_ and *φ*_*SEP*_, parametrized by the phenotypic effect between any genotype g (combination of wild-type, heterozygous or homozygous mutants) and the wild-type $\theta_{g}^{\text{PLT}}$ and $\theta_{g}^{\text{SEP}}$ at the PLTs or SEP pair of loci, respectively:

$$\varphi_{\text{PLT}}\left(x_{i}\right)=\sum_{g\neq \text{WT}}\theta_{g}^{\text{PLT}}s_{g}^{\text{PLT}}\left(x_{i}\right),\;\varphi_{\text{SEP}}\left(x_{i}\right)=\sum_{g\neq \text{WT}}\theta_{g}^{\text{SEP}}s_{g}^{\text{SEP}}\left(x_{i}\right),$$

where $s_{g}^{\text{PLT}}\left(x_{i}\right)$ and $s_{g}^{\text{PLT}}\left(x_{i}\right)$ take value 1 if the genotype at the PLT or SEP loci match g and 0 otherwise. Note that *φ*_*PLT*_ takes a different value for every possible combination of mutations in *PLT3* and *PLT7* and *φ*_*SEP*_ takes a different value for every possible combination of mutations in *EJ2* and *J2*, so that inferring the values for *φ*_*PLT*_ and *φ*_*SEP*_ is equivalent to allowing a full set of additive, dominance and pairwise interactions within each of *PLT3/PLT7* and *EJ2*/*J2*. These two pairwise models are then combined through a multilinear function into the log-transformed average expected number of branching events $\mu \left(x_{i}\right)$, given by

$$\mu_{\text{hierarchical}}\left(x_{i}\right)=\theta_{\text{WT}}+\varphi_{\text{PLT}}\left(x_{i}\right)+\varphi_{\text{SEP}}\left(x_{i}\right)-\theta_{\text{Int}}\varphi_{\text{PLT}}\left(x_{i}\right)\varphi_{\text{SEP}}\left(x_{i}\right),$$

where *θ*_WT_ is the wild-type log-transformed expected branching events, *φ*_*PLT*_ controls the log effect of the relevant combination of mutations in *PLT3* and *PLT7* when placed in a wild-type *EJ2 J2* background, *φ*_*SEP*_ controls the log effect of the relevant combination of mutations in *EJ2* and *J2* combinations in a wild-type *PLT3 PLT7* background and *θ*_Int_ represents the masking interaction between the two phenotypes^36^. Finally, as in the standard linear models from the previous section, the observed number of branching events $y_{i}$ for a plant i with genotype $x_{i}$ and $t_{i}$ inflorescences is drawn from a negative binomial distribution with overdispersion parameter α as in the linear models from the previous section:

$$y_{i}\sim \text{NegativeBinomial}\left({t_{i}\times e}^{\mu_{\text{hierarchical}}\left(x_{i}\right)},\alpha \right).$$

In summary, the hierarchical model is equivalent to fitting pairwise interactions within paralog pairs, then combining the within pair effects via a multilinear interaction across pairs, and then transforming the result via an exponential function. Extended Data Fig. 4a shows a graphical representation of the complete model. This model was coded in PyTorch^70^ and the maximum likelihood solution was found running the Adam optimizer for 10,000 iterations and checking for convergence. Extended Data Fig. 4c shows the inferred model including all the *EJ2*^*pro*^ alleles and illustrates how the different layers of the hierarchical model are applied and combined together to predict the expected number of branching events for a given genotype.

## Extended Data

## Supplementary Material

Supplementary_Information_Figures **Supplementary Figure 1: Presence of *EJ2* and *J2* in the Solanaceae.** Left: Phylogenetic tree of 23 species of the genus *Solanum*. Scale bar represents branch length in coalescent units. Right: Copy number of *EJ2* and *J2* genes showing EJ2 present in single copy (light blue) and *J2* varying between absent (grey), single copy (light blue) and two copies (dark blue). Figure and data adapted from Benoit *et al*.^4^
**Supplementary Figure 2: Additional *EJ2 cis*-regulatory alleles. (a)** A 6 kb region upstream of the *EJ2* transcription start site showing open chromatin (blue), conserved non-coding sequences (CNSs, dark blue), predicted transcription factor binding sites (TFBSs, orange), and pan-genome variants (light orange). Three regions targeted by CRISPR/Cas9 editing in this study are highlighted in light blue, pink, and light green. **(b)** Left: 153 bp target region located 5.7kb upstream of the *EJ2* transcription start site showing annotated TFBSs (orange), including the focal DOF and AP2/ERF binding sites. Also shown is a CRISPR/dCas9-ABE8E base editor gRNA (light blue) used for targeted mutagenesis to generate an allelic series (*EJ2*^*pro*^). SNVs, blue. Right: Quantifications of inflorescence branching for each genotype. Area of grey circles, numbers of inflorescences; black diamonds and bars, mean values and standard deviations. Total number (n) of observations and adjusted p-values from Two-sided Dunnett’s Compare with Control Test. **(c)** Left: 225 bp target region located 1.6kb upstream of the *EJ2* transcription start site showing CRISPR gRNA (pink) used for targeted mutagenesis to generate an allelic series (*EJ2*^*pro*^). Dashed red lines, deleted sequences; Deletion sizes and SNVs, blue. Right: Quantifications of inflorescence branching for each genotype. Area of grey circles, numbers of inflorescences; black diamonds and bars, mean values and standard deviations. Total number (n) of observations and adjusted p-values from Two-sided Dunnett’s Compare with Control Test. **(d)** Left: 232 bp target region located 2.1kb upstream of the *EJ2* transcription start site showing annotated TFBSs (orange). Also shown is a CRISPR gRNA (light green) used for targeted mutagenesis to generate an allelic series (*EJ2*^*pro*^). Dashed red lines, deleted sequences; Deletion sizes and SNVs, blue. Right: Quantifications of inflorescence branching for each genotype. Area of grey circles, numbers of inflorescences; black diamonds and bars, mean values and standard deviations. Total number (n) of observations and adjusted p-values from Two-sided Dunnett’s Compare with Control Test. **(e)** Representative images of *j2* (same as Fig. 2) and four phenotypic *EJ2*^*pro*^ alleles in the *j2* background, capturing the range of branching effects. Red arrowheads mark branch points. Scale bars are 1 cm.
**Supplementary Figure 3: Fruit shape and sepal length phenotypes of *EJ2 cis*-regulatory alleles. (a)** Transverse sections of representative mature green fruit. Scale bar, 1 cm. **(b)** Ratio of maximum height to width of mature green fruits (Fruit Shape Index I)^63^. Grey circles, index, scaled by number of fruits; black diamonds and bars, mean values and standard deviations. Total number (n) of fruits and adjusted p-values from Two-sided Dunnett’s Compare with Control Test. **(c)** Ratio of sepal length allele/*j2* background. Grey circles, ratio, scaled by number of flowers; black diamonds and bars, mean values and standard deviations. Total number (n) of flowers and adjusted p-values from Two-sided Dunnett’s Compare with Control Test.
**Supplementary Fig. 4: *DOF9* transcription factor mutant has no phenotype in inflorescence architecture. (a)** Expression dynamics of *EJ2, J2, DOF9*, and *DOF2* over five developmental stages (depicted in grey): Early Vegetative Meristem (EVM), Middle (MVM), Late (LVM), Transition (TM), and Floral Meristem (FM). Despite similar expression dynamics, *DOF2* is lowly expressed (9.06 transcripts per million (TPM) in LVM, lower than associated LVM leaf primordia samples at 25.25 TPM)^34^. **(b)** Quantification of branching in *dof9* mutants. Area of grey circles correspond to the number of inflorescences, black diamonds are mean values and bars are standard deviations. Number (n) of observations and p-values from Two-sided Dunnett’s Compare with Control Test shown in blue. **(c)** Representative inflorescences of WT and *dof9* mutant. Scale bar is 1 cm. **(d)** Representative fifth leaves of WT and *dof9* mutants. Scale bar is 1 cm.
**Supplementary Figure 5: Models for the number of branching events. (a)** Scatterplot of the per-plant average number of branching events and the variance across inflorescences within the same plant. Dashed line shows expectation under the assumption that branching events are Poisson distributed. **(b)** Scatterplot of the per-genotype and season average number of branching events and the variance across inflorescences within the same genotype and season. Dashed line shows expectation under the assumption that branching events are Poisson distributed. **(c)** Scatterplot showing the predicted branching events under an additive negative binomial regression model with identity link function and observed per-plant average number of branching events. **(d)** Scatterplot showing the predicted branching events under an additive negative binomial regression model with log link function (i.e. assuming that mutations combine multiplicatively) and observed per-plant average number of branching events.
**Supplementary Figure 6: Predictive performance of additive, pairwise and hierarchical model on held-out seasons and genotypes.** Scatterplots comparing the log expected number of branching events under different models with the log Maximum Likelihood Estimate (MLE) of the genotype-season means for different subsets of held-data. In each of the first 4 columns data from a single season is held-out, whereas in the last column the same random 10% of genotypes are held-out across all seasons. Error bars represent the 95% confidence interval for the MLEs. Genotype-season combinations with a 95% confidence interval wider than a thousand-fold range are not shown. The reported R^2^ values correspond to the squared Pearson coefficients between the log predicted number of branches and the log MLEs for the held-out data.

Supplementary_Tables

The online version contains supplementary material available at https://doi.org/10.1038/s41586-025-09243-0

## Figures and Tables

**Table 1.** Estimates of fold change relative to the expectation under the multiplicative model for combinations of perturbations within each paralog pair under the pairwise interaction model. P-values from two-sided z-tests for the specified contrasts estimated after fitting a pairwise interaction model with negative binomial likelihood using *statsmodels*.^69^ P values are reported in the figure panels for figure(s) 1d–e,2a,3d. For the full set of estimated parameters, see Supplementary Table 21.

Genotype	Fold Change in Excess of Multiplicative Expectation	95% Confidence interval	P-value
*plt3/+ plt7*	3.29	[2.64, 4.10]	1.44·10^−26^
*plt3 plt7/+*	3.27	[2.65, 4.04]	2.95·10^−28^
*EJ2*^*pro3*^ *j2*	6.81	[4.21, 11.03]	5.16·10^−15^
*EJ2*^*pro4*^ *j2*	9.32	[5.25, 16.57]	2.74·10^−14^
*EJ2*^*pro1*^ *j2*	7.05	[5.21, 9.55]	9.91·10^−37^
*EJ2*^*pro7*^ *j2*	16.97	[8.14, 35.38]	4.16·10^−14^
*EJ2*^*pro8*^ *j2*	5.95	[4.42, 8.03]	1.13·10^−31^
*EJ2*^*pro6*^ *j2*	6.49	[4.56, 9.23]	3.10·10^−25^

## Data Availability

All data are available within this Article and its Supplementary Information. RNA sequencing data generated in this study is available at the Gene Expression Omnibus (https://www.ncbi.nlm.nih.gov/geo/) under accession GSE289537, and previously published RNA sequencing is available at Sequence Read Archive (SRA, https://ncbi.nlm.nih.gov/sra) under BioProjects PRJNA376115 and PRJNA343677. The tomato pangenome is available at SRA under BioProject PRJNA557253. The Arabidopsis thaliana non-redundant motif database used is available at https://planttfdb.gao-lab.org/index.php?sp=Ath. The raw data with the number of branching events for each plant and inflorescence is provided in Supplementary Tables. All unique biological materials used in this manuscript are available for distribution upon request.

## Code availability

Code to reproduce the statistical analysis, quantitative modeling and the derived Figures is available at https://github.com/cmarti/tomato_branching/tree/master, DOI: 10.5281/zenodo.15552524.
