# Supplementary material for: Cryptic variation fuels plant phenotypic change through hierarchical epistasis
Source: Nature. 2025 Jul 9;644(8078):984–92. doi: 10.1038/s41586-025-09243-0 (PMC12282530; doi:10.1038/s41586-025-09243-0)
Supplement: Supplementary file 1 — Supplementary Figs. 1–6. [file 41586_2025_9243_MOESM1_ESM.pdf]

nature portfolio

https://doi.org/10.1038/s41586-025-09243-0

Supplementary information

Cryptic variation fuels plant phenotypic change through hierarchical epistasis

In the format provided by the
authors and unedited

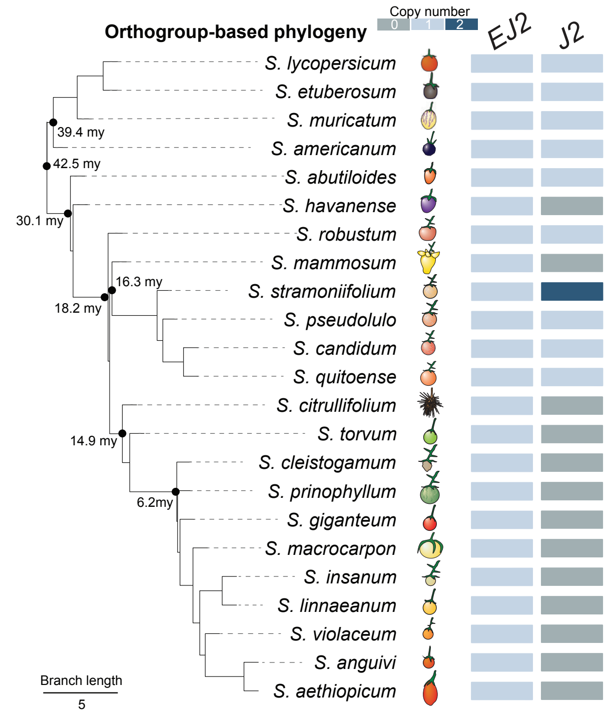

Supplementary Figure 1: Presence of *EJ2* and *J2* in the *Solanaceae*. Left: Phylogenetic tree of 23 species of the genus *Solanum*. Scale bar represents branch length in coalescent units. Right: Copy number of *EJ2* and *J2* genes showing *EJ2* present in single copy (light blue) and *J2* varying between absent (grey), single copy (light blue) and two copies (dark blue). Figure and data adapted from Benoit *et al*.[4]

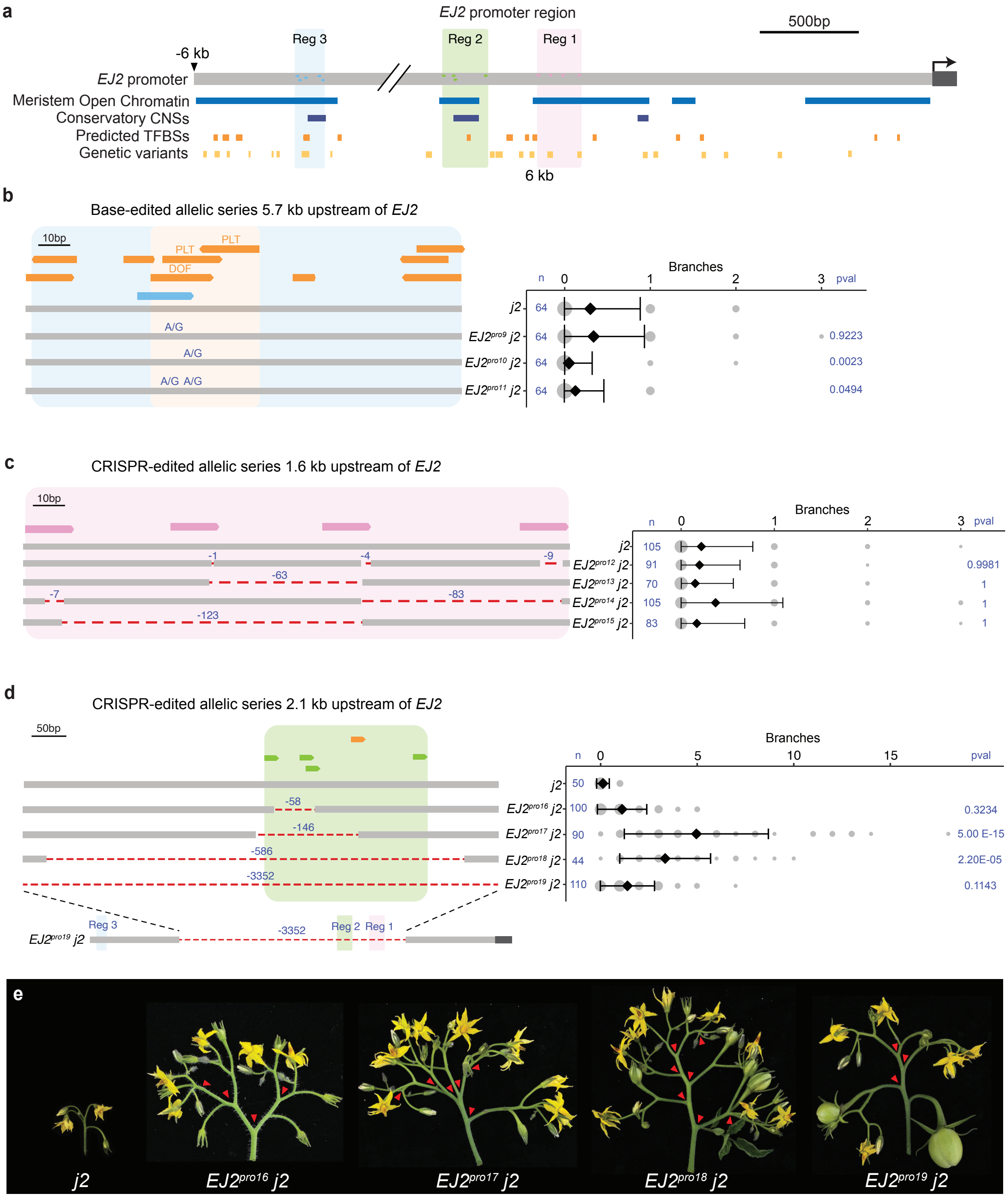

Supplementary Figure 2: Additional *EJ2* cis-regulatory alleles. (a) A 6 kb region upstream of the *EJ2* transcription start site showing open chromatin (blue), conserved non-coding sequences (CNSs, dark blue), predicted transcription factor binding sites (TFBSs, orange), and pan-genome variants (light orange). Three regions targeted by CRISPR/Cas9 editing in this study are highlighted in light blue, pink, and light green. **(b)** Left: 153 bp target region located 5.7kb upstream of the *EJ2* transcription start site showing annotated TFBSs (orange), including the focal DOF and AP2/ERF binding sites. Also shown is a CRISPR/dCas9-ABE8E base editor gRNA (light blue) used for targeted mutagenesis to generate an allelic series (*EJ2pro*). SNVs, blue. Right: Quantifications of inflorescence branching for each genotype. Area of grey circles, numbers of inflorescences; black diamonds and bars, mean values and standard deviations. Total number (n) of observations and adjusted p-values from Two-sided Dunnett's Compare with Control Test. **(c)** Left: 225 bp target region located 1.6kb upstream of the *EJ2* transcription start site showing CRISPR gRNA (pink) used for targeted mutagene-sis to generate an allelic series (*EJ2pro*). Dashed red lines, deleted sequences; Deletion sizes and SNVs, blue. Right: Quantifications of inflorescence branching for each genotype. Area of grey circles, numbers of inflorescences; black diamonds and bars, mean values and standard deviations. Total number (n) of observations and adjusted p-values from Two-sided Dunnett's Compare with Control Test. **(d)** Left: 232 bp target region located 2.1kb upstream of the *EJ2* transcription start site showing annotated TFBSs (orange). Also shown is a CRISPR gRNA (light green) used for targeted mutagenesis to generate an allelic series (*EJ2pro*). Dashed red lines, deleted sequences; Deletion sizes and SNVs, blue. Right: Quantifications of inflorescence branching for each genotype. Area of grey circles, numbers of inflorescences; black diamonds and bars, mean values and standard deviations. Total number (n) of observations and adjusted p-values from Two-sided Dunnett's Compare with Control Test. **(e)** Representative images of *j2* (same as Fig. 2) and four phenotypic *EJ2pro* alleles in the *j2* background, capturing the range of branching effects. Red arrowheads mark branch points. Scale bars are 1 cm.

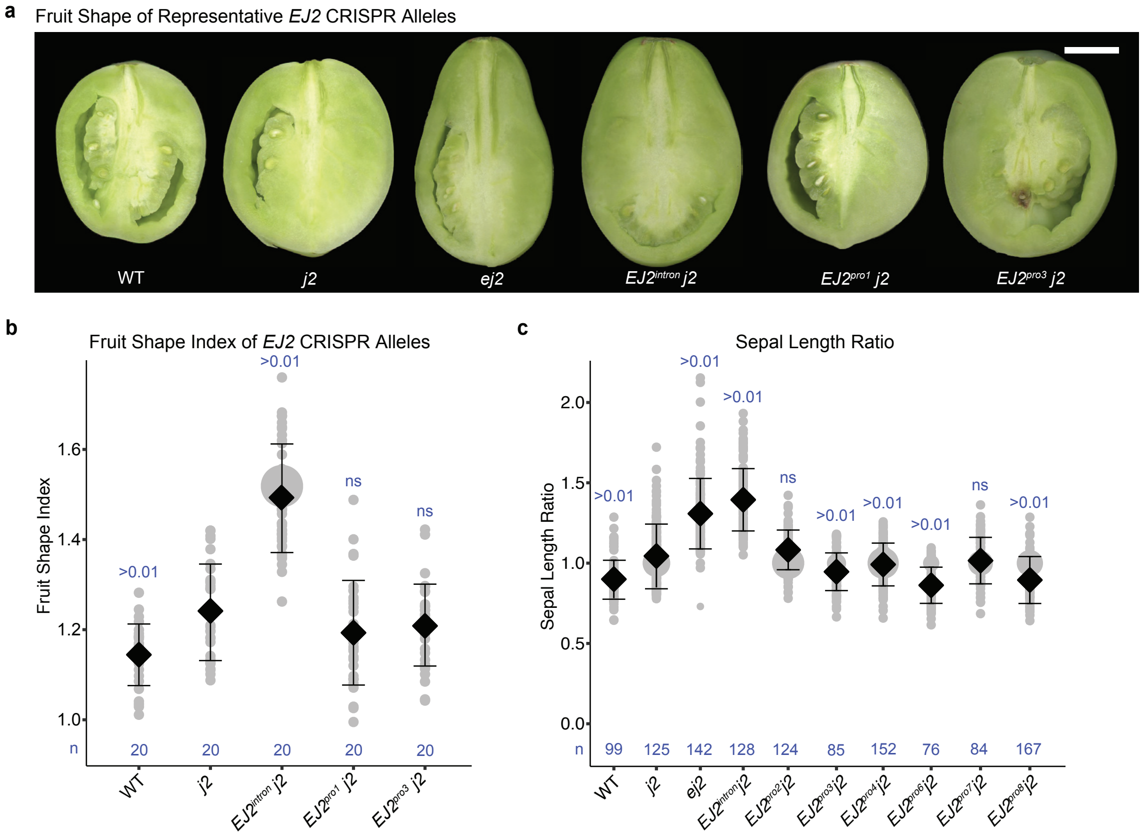

Supplementary Figure 3: Fruit shape and sepal length phenotypes of *EJ2* cis-regulatory alleles. (a) Transverse sections of representative mature green fruit. Scale bar, 1 cm. **(b)** Ratio of maximum height to width of mature green fruits (Fruit Shape Index I)[63]. Grey circles, index, scaled by number of fruits; black diamonds and bars, mean values and standard deviations. Total number (n) of fruits and adjusted p-values from Two-sided Dunnett's Compare with Control Test. **(c)** Ratio of sepal length allele/*j2* background. Grey circles, ratio, scaled by number of flowers; black diamonds and bars, mean values and standard deviations. Total number (n) of flowers and adjusted p-values from Two-sided Dunnett's Compare with Control Test.

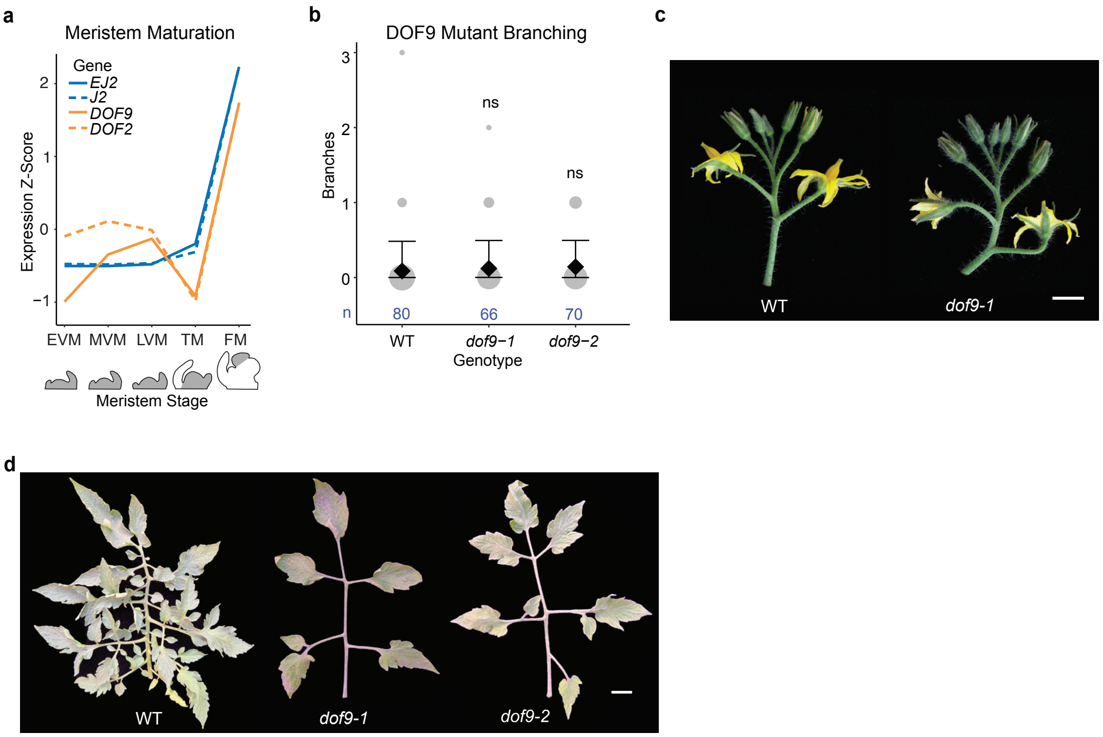

Supplementary Fig. 4: *DOF9* transcription factor mutant has no phenotype in inflorescence architecture. (a) Expression dynamics of *EJ2*, *J2*, *DOF9*, and *DOF2* over five developmental stages (depicted in grey): Early Vegetative Meristem (EVM), Middle (MVM), Late (LVM), Transition (TM), and Floral Meristem (FM). Despite similar expression dynamics, *DOF2* is lowly expressed (9.06 transcripts per million (TPM) in LVM, lower than associated LVM leaf primordia samples at 25.25 TPM)[34]. **(b)** Quantification of branching in dof9 mutants. Area of grey circles correspond to the number of inflorescences, black diamonds are mean values and bars are standard deviations. Number (n) of observations and p-values from Two-sided Dunnett's Compare with Control Test shown in blue. **(c)** Representative inflorescences of WT and *dof9* mutant. Scale bar is 1 cm. **(d)** Representative fifth leaves of WT and *dof9* mutants. Scale bar is 1 cm.

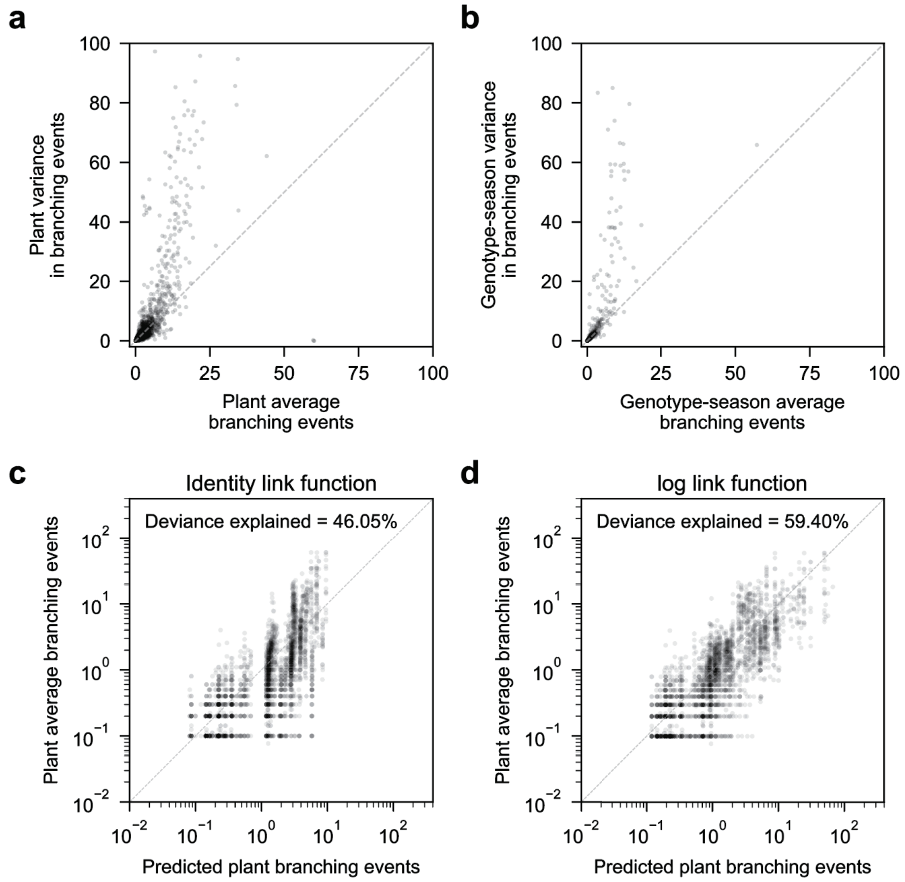

Supplementary Figure 5: Models for the number of branching events. (a) Scatterplot of the per-plant average number of branching events and the variance across inflorescences within the same plant. Dashed line shows expectation under the assumption that branching events are Poisson distributed. **(b)** Scatterplot of the per-genotype and season average number of branching events and the variance across inflorescences within the same genotype and season. Dashed line shows expectation under the assumption that branching events are Poisson distributed. **(c)** Scatterplot showing the predicted branching events under an additive negative binomial regression model with identity link function and observed per-plant average number of branching events. **(d)** Scatterplot showing the predicted branching events under an additive negative binomial regression model with log link function (i.e. assuming that mutations combine multiplicatively) and observed per-plant average number of branching events.

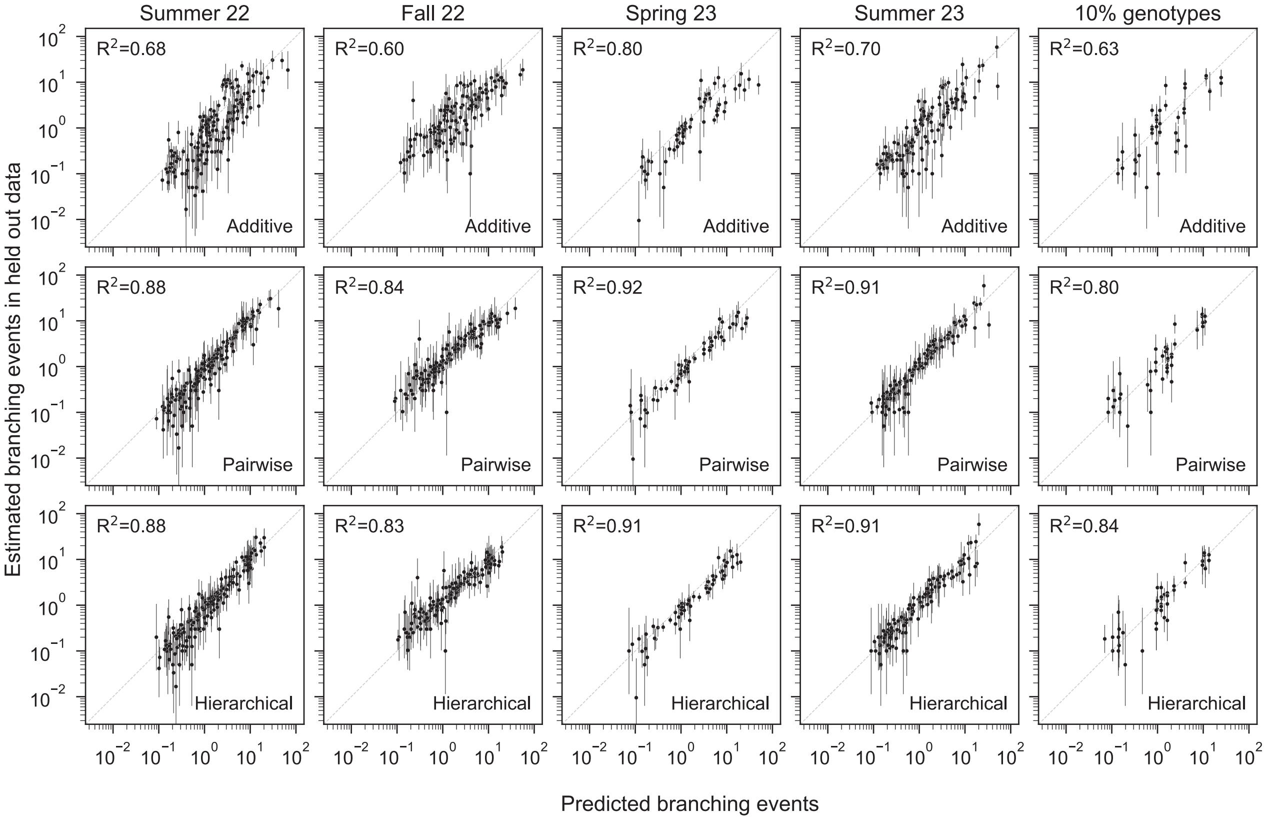

Supplementary Figure 6: Predictive performance of additive, pairwise and hierarchical model on held-out seasons and genotypes. Scatterplots comparing the log expected number of branching events under different models with the log Maximum Likelihood Estimate (MLE) of the genotype-season means for different subsets of held-out data. In each of the first 4 columns data from a single season is held-out, whereas in the last column the same random 10% of genotypes are held-out across all seasons. Error bars represent the 95% confidence interval for the MLEs. Genotype-season combinations with a 95% confidence interval wider than a thousand-fold range are not shown. The reported R^2 values correspond to the squared Pearson coefficients between the log predicted number of branches and the log MLEs for the held-out data.
